# Human and machine validation of 14 databases of dynamic facial expressions

**DOI:** 10.3758/s13428-020-01443-y

**Published:** 2020-08-17

**Authors:** Eva G. Krumhuber, Dennis Küster, Shushi Namba, Lina Skora

**Affiliations:** 1 Department of Experimental Psychology, University College London, 26 Bedford Way, London, WC1H 0AP UK; 2 University of Bremen, Bremen, Germany; 3 Jacobs University Bremen, Bremen, Germany; 4 Hiroshima University, Hiroshima, Japan; 5 Present Address: School of Psychology, University of Sussex, Brighton, UK

**Keywords:** Facial expression, Emotion, Dynamic, Database, Machine analysis, FACS

## Abstract

With a shift in interest toward dynamic expressions, numerous corpora of dynamic facial stimuli have been developed over the past two decades. The present research aimed to test existing sets of dynamic facial expressions (published between 2000 and 2015) in a cross-corpus validation effort. For this, 14 dynamic databases were selected that featured facial expressions of the basic six emotions (anger, disgust, fear, happiness, sadness, surprise) in posed or spontaneous form. In Study 1, a subset of stimuli from each database (*N* = 162) were presented to human observers and machine analysis, yielding considerable variance in emotion recognition performance across the databases. Classification accuracy further varied with perceived intensity and naturalness of the displays, with posed expressions being judged more accurately and as intense, but less natural compared to spontaneous ones. Study 2 aimed for a full validation of the 14 databases by subjecting the entire stimulus set (*N* = 3812) to machine analysis. A FACS-based Action Unit (AU) analysis revealed that facial AU configurations were more prototypical in posed than spontaneous expressions. The prototypicality of an expression in turn predicted emotion classification accuracy, with higher performance observed for more prototypical facial behavior. Furthermore, technical features of each database (i.e., duration, face box size, head rotation, and motion) had a significant impact on recognition accuracy. Together, the findings suggest that existing databases vary in their ability to signal specific emotions, thereby facing a trade-off between realism and ecological validity on the one end, and expression uniformity and comparability on the other.

**Electronic supplementary material:**

The online version of this article (10.3758/s13428-020-01443-y) contains supplementary material, which is available to authorized users.

## Introduction

The human face is an important source of dynamic information. By conveying rich and complex action patterns, the dynamic quality of facial behavior makes it a powerful medium for emotion communication. Yet, for years the majority of research on the visual perception of emotions was dominated by static stimuli, i.e. datasets of still images of emotional expressions captured at apex (e.g., Ekman & Friesen, 1976; Biehl et al., 1997; Goeleven et al., 2008; Tottenham et al., 2009). Apart from their questionable ecological validity which renders them untypical of the displays encountered in everyday life (Russell, 1994), static portrayals may not convey the same affective information and communicative intent. There is now a growing body of evidence suggesting that the dynamics of facial expressions are crucial for the recognition (e.g., Wehrle et al., 2000; Kamachi et al., 2001) and interpretation of emotions (e.g., Ambadar et al., 2005; see Krumhuber et al., 2013; Sato et al., 2019 for reviews). Moreover, moving stimuli elicit different patterns of muscular/behavioral responses (Sato & Yoshikawa, 2007) and brain activation compared to static ones (Zinchenko et al., 2018). In order to capture the actual form of human behavior, facial movement appears to be essential for an accurate approximation of reality. In this vein, the last two decades have seen increased questioning and criticism of static stimuli, and a gradual shift towards research on dynamic expressions.

To meet new demands in stimulus selection that reflect the dynamic quality of facial displays, a wide range of databases have been developed in recent years. Those largely vary in their scope and potential application. Furthermore, they employ a host of techniques for expression elicitation. In some databases, for example, subjects are asked to deliberately make an expression by activating certain facial muscles using the Directed Facial Action task (Ekman, 2007). Alternatively, acting techniques have been used for simulating the emotion by asking subjects to (re)produce a particular emotion. This may involve the use of labels or verbally rich scenarios (so-called vignettes) that specify the emotional content (Siedlecka & Denson, 2019). In a few databases, expressions are also elicited through mental imagery in which the person recalls a personal past event and subsequently enacts the relevant emotion using Stanislavski or method acting techniques (Scherer & Bänzinger, 2010). While portrayals of the latter type may contain experiential affective elements, they are displayed with the deliberate intent to communicate the desired emotion. Hence, all of the above methods can be summarized under the umbrella of posed expression elicitation. A different approach consists in capturing spontaneous expressions by exposing naïve subjects to events expected to evoke a particular emotional state. These can be active tasks such as playing video games or touching certain objects (Cowie et al., 2005). Alternatively, databases may rely on emotion-induction techniques that are more passive such as watching emotive pictures, movies, or listening to music (Coan & Allen, 2007). Here, subjects respond freely and in their own way, yet the induced emotional expressions occur in a controlled setting (often in the laboratory).

Up to now, most of the available dynamic databases have favored some variant of posing over spontaneous emotion elicitation. Deliberately posed expressions can be defined precisely and judged against a clear criterion set by the researcher. However, they have been argued to represent stereotypical and often exaggerated displays (Barrett, 2011). Because acted portrayals operate with an explicit intention to convey the necessary facial signals, they are of higher expressivity compared to spontaneous emotional expressions (Hess et al., 1997). These differences are reflected in the cortical innervation of the underlying facial muscles, implying two separate neural pathways for voluntary and involuntary actions (i.e., cortical and subcortical, Morecraft et al., 2001; Rinn, 1984). Supportive evidence comes from studies showing that posed expressions have different temporal and morphological characteristics (duration, intensity, asymmetry) than spontaneous ones (Cohn & Schmidt, 2004; Krumhuber & Manstead, 2009; Namba et al., 2017). Databases in which emotions were spontaneously induced may therefore feature more salient facial behavior, which might guide recognition accuracy. In this vein, emotion agreement was found to be lower and vary substantially across spontaneous expressions, ranging from 15% to 65% (Wagner, 1990; Kayyal & Russell, 2013; for a review see Calvo & Nummenmaa, 2016). By contrast, recognition rates are typically situated between 60% and 80% for posed expressions. While this evidence points toward generally weaker recognizability for spontaneous compared to posed facial expressions, existing findings are difficult to interpret.

Many studies have tested their own database without any comparative evaluations between different platforms. Hence, the validity of conclusions about emotion decoding accuracy depends on the specific stimulus set being used. Furthermore, study authors have utilized dissimilar procedures to assess recognition performance. For the evaluation of some databases, for example, judgment tasks have been used in which trained raters or lay observers selected an emotion label from a predetermined list of categories (varying between 6 and 24; Golan et al., 2006; Roy et al., 2007). Others have calculated interrater agreement on the emotion categories among small groups of people, often experts or annotators (Zhang et al., 2014). Besides a strict categorical approach, a few databases have obtained emotion confidence and/or intensity judgments, continuous emotion ratings, or employed open-response formats (Kaulard et al., 2012; Matuszewski et al., 2012; Meillon et al., 2010). Alternative measures have included self-reports of emotional experience, thereby relying on subjective self-assessments instead of observer-based ones (Barrett, 2006). Finally, component measures have focused on the analysis of facial actions (using the Facial Action Coding System (FACS), Ekman et al., 2002) to obtain an objective classification of the expressive behavior (Cosker et al., 2011).

Given the various methods employed for eliciting and validating dynamic facial expressions, the quantity and quality of data available on emotion recognition performance is a major issue (Küster et al., 2020). There is currently no normative standard that incorporates the diversity of approaches seen in the literature. This calls for common cross-corpus evaluations that make it possible to compare databases to each other. Such coordinated effort may help accelerate the progress in the field by providing researchers with a benchmark by which to review, compare, and contrast existing study findings. Having a comprehensive source of reference provides crucial insights into human performance and how that varies within and across databases. Moreover, it is essential for the measurement and classification of emotions by means of machine learning.

In the last two decades, significant advances have been made in automated affect recognition (Sandbach et al., 2012), including the development of commercial software for dynamic facial expression analysis. The ability to recognize a person’s expression automatically and in real-time offers unique opportunities in basic and applied research (Zeng et al., 2009). However, many systems so far have been trained and tested on limited sets of data (Pantic & Bartlett, 2007). Those typically involved posed or acted facial behavior displaying prototypical patterns of emotional expression. In this vein, machine classification performance was found to be high for deliberately posed stimuli (Beringer et al., 2019; Skiendziel et al., 2019), but was reduced when facial expressions were spontaneous and/or subtle in their appearance (Yitzhak et al., 2017; Krumhuber et al., 2020). Unless training sets encompass large stimulus collections, automatic systems may therefore fail to generalize to the wide variety of expressive displays common in everyday life.

### The present research

This research aims to provide a comparative test of databases of dynamic facial expressions published between 2000 and 2015. Such cross-corpus investigation allows for the comparison and validation of dynamic stimuli that differ in a range of parameters (i.e., elicitation condition, gender, ethnicity, expression intensity, head pose). All selected sets are publicly available and feature basic emotions in visual format. A comprehensive review of the existing corpora in terms of their conceptual and practical features is given in Krumhuber et al. (2017). In the present paper, we focus on the empirical evaluation by measuring and comparing emotion recognition indices across individual databases. For this purpose, we collected data from human observers and conducted automated facial expression analysis with a software tool called FACET (iMotions). FACET has been used widely, thereby demonstrating superior levels of emotion classification in recent cross-classifier comparisons (Stöckli et al., 2018; Dupré et al., 2020).

In Study 1, human participants were presented with a subset of stimuli from 14 dynamic databases, yielding facial expressions of the basic six emotions that were either posed or spontaneous. Recognition performance was assessed through an emotion identification task, including ratings of expression intensity and naturalness. We also submitted the materials to automated analysis by means of FACET as an additional form of validation, and to compare the results of the machine analysis to human coding. Given the diversity of expressive stimuli in this broad set of databases, we expected considerable variance in classification accuracy across the databases. Recognition levels should further vary with the perceived intensity and naturalness of the displays, with posed expressions being judged more accurately and as intense, but less natural compared to spontaneous ones.

Study 2 aimed for a full validation of the 14 databases by subjecting the entire stimulus sets to automated analysis by means of FACET. We further examined the exact facial cues that contribute to expression recognition by conducting a FACS-based Action Unit (AU) analysis. Similar to the first study, posed expressions were expected to facilitate emotion classification, thereby exhibiting prototypical facial AU configurations. Prototypicality should in turn predict accuracy in emotion identification, with increasingly better performance expected for more prototypical expressions. Aside from an emotion-based analysis, we examined the technical features of each database (i.e., duration, face box size, head rotation and motion), and their impact on recognition accuracy. While smaller face sizes and larger head movements may pose a more challenging situation, longer video durations could positively affect machine classification.

## Study 1

The aim of the first study was to provide initial validation results for a subset of stimuli from each of the 14 dynamic databases. To this end, human observers were asked to identify the expressed emotion as well as to rate the intensity and naturalness for each stimulus. We further obtained machine validation data on the same materials using commercial software for automated affect analysis.

### Method

#### Materials

Given the practical limitations regarding the number of facial portrayals that could be rated by human observers, a subset of stimuli was selected from the 14 databases using stratified random sampling. All contained videos of dynamic facial expressions portrayed by individual encoders and featured basic emotions. Out of the 14 databases, nine showed posed facial expressions that were initiated via instructions to perform an expression/facial action or through scenario enactment techniques: ADFES, BU-4DFE, CK, D3D-FACS, DaFEx, GEMP, MMI, MPI, and STOIC. The other five databases featured spontaneous facial expressions that were elicited in response to videos or tasks designed to induce a specific emotion: BINED, DISFA, DynEmo, FG-NET, UT Dallas. Both types of expressions had been recorded in the laboratory by the database authors. For the purpose of this research, we focused on the following six basic emotions as predefined by the dataset authors: anger disgust, fear, happiness, sadness, and surprise.^1^

For every database, two exemplars were randomly selected from each emotion category, yielding 12 portrayals per database. The two exceptions were DISFA and DynEmo, both of which contain only five and four basic emotions, respectively. This yielded a total of 162 expressions (108 posed, 54 spontaneous) from 85 female and 77 male encoders. Portrayals that exceeded a duration of 15 s (BINED, DynEmo) were edited to display the dynamic trajectory from onset, over apex, to offset of the expression (if applicable). The final stimuli lasted on average 5 s and measured approximately 642 x 482 pixels.

### Human observers

#### Participants

One hundred twenty-four participants (86 females), aged 18–45 years (M = 24.23, SD = 5.58) were recruited face-to-face or via the departmental subject pool and participated in exchange for course credit or payment of £6. All participants identified themselves as White Caucasian. A power analysis using G*Power 3.1 (Faul et al., 2007) indicated that this sample size is sufficient to detect a medium-sized effect of database or emotion (Cohen’s *f* = 0.25) in an ANOVA with 80% statistical power (α = 0.05). All participants provided written informed consent prior to the study. Ethical approval was granted by the Department of Experimental Psychology at University College London.

#### Procedure

The study was described as a test of how people perceive emotion in dynamic facial expressions, with all instructions and stimuli being presented via computer. Participants saw one out of two exemplars of each emotion category from every database, netting 81 dynamic facial expressions per participant.^2^ Stimulus sequence was randomized using Qualtrics (Provo, UT). For each facial stimulus, participants rated their confidence (from 0 to 100%) about the extent to which the expression reflects anger, disgust, fear, happiness, sadness, surprise, other emotion, and neutral (no emotion). If they felt that more than one category applied, they could respond using multiple sliders to choose the exact confidence levels for each response category. Ratings across the eight response options had to sum up to 100%. In addition, participants evaluated each facial stimulus in terms of its intensity and naturalness of the expressed emotion, using 7-point Likert scales (1 - *very weak*, 7 - *very intense*; 1 - *not natural at all*, 7 - *very natural*). All three measures were presented on the same screen and in a fixed order, with unlimited response time.

### Machine analysis

We submitted all video stimuli to automated analysis by means of the FACET classifier, which is part of the biometric software suite by iMotions (www.imotions.com, SDK v6.3). FACET is a commercial software for automatic facial expression measurement based on the Computer Expression Recognition Toolbox algorithm (CERT; Littlewort et al., 2011). It estimates facial expressions in terms of the six basic emotions as well as 20 FACS Action Units (AUs). FACET outputs per-frame evidence scores for each emotion category that represent estimates of the likelihood of an expert human coder recognizing the expression as the target category. The values are expressed on a decimal logarithmic scale centered around zero (similar to a z-score), with zero indicating a 0.5 probability, negative values indicating that an expression is likely not present, and positive values indicating the likely presence of an expression.

Importantly, these raw evidence scores do not include any specification in terms of which emotion is most probable relative to the other emotions. Hence, researchers interested in dynamic expressions need to define a metric or rule by which to aggregate the per-frame evidence, and to extract the dominant emotion categorization for each video stimulus (Dente et al., 2017). While FACET’s raw evidence scores can be averaged to determine the dominant emotion categorization (e.g., Yitzhak et al., 2017), this approach results in a linear “pooling” of evidence across frames, with probabilities that may no longer reflect the logarithmically scaled recognition odds provided by human experts. We therefore transformed the FACET raw, non-baseline-corrected, evidence values first into probabilities, using the formula provided in the FACET documentation (1/(1 + (10 ^ -evidence); iMotions, 2016), and then into odds values (1/((1/*p*)-1)). Such conversion on a scale from zero to infinity ensures that the logarithmic increase in probabilities produced by the binary classifiers is adequately reflected when averaging across all frames. We defined the dominant emotion categorization as the expression with the highest proportion of odds relative to the total amount of odds for all six basic expressions:

$$ {\mathrm{Confidence}}_E=\frac{\sum_{i=1}^n{x}_i}{\sum_{i=1}^n{x}_i+{\sum}_{i=1}^n{y}_i}\ast 100 $$

For each expression (E), we computed a *confidence* score reflecting the proportion of the summed odds for the expression (x) relative to the total of all odds (target expression (x) + other expressions (y)). This proportion (0-1) was subsequently converted into a percentage score by multiplying the value with 100. This approach yields an odds-based percentage score for each video that allows easy identification of the dominant emotion categorization, i.e., the category with the highest score. Additionally, it provides a simple standardized metric to quantify and rank the relative confidence for each expression across videos from diverse databases. By definition, the resulting confidence scores for each expression add up to a total of 100.

To compute the human and machine accuracy of the multi-class categorization, we created new dummy variables to indicate the recognized expressions, and whether they matched the predicted emotion labels (true vs. false).

### Results

Rating scores were averaged across the two exemplars of each emotion category from every database, which served as the unit of analysis. For all analyses, a Greenhouse-Geisser adjustment to degrees of freedom was applied, and Bonferroni correction was used for multiple comparisons.

#### Emotion recognition

Recognition accuracy was significantly higher than chance (17%), in both humans, M = 65.11% (SD = 26.18), *t*(80) = 16.54, *p* < .001, Cohen’s *d* = 1.84, and machine, M = 65.43% (SD = 40.03), *t*(80) = 10.89, *p* < .001, Cohen’s *d* = 1.21. In general, expressions from posed datasets were better recognized than those from spontaneous ones in both humans, *t*(38.39) = 3.64, *p* = .001, Cohen’s *d* = .91, and machine, *t*(79) = 2.21, *p* = .030, Cohen’s *d* = .50.

**Fig. 1:**
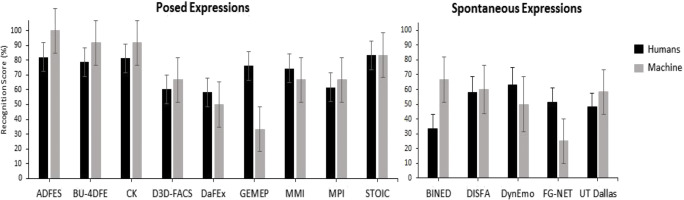
Mean recognition rates of posed and spontaneous expressions per database by human observers vs. machine classifier in Study 1. Error bars represent SEM

Due to insufficient variance within the study cells, separate ANOVAs were conducted with the factors database (14) or emotion (6), thereby comparing human vs. machine performance. Results revealed a significant main effect of database, *F*(13, 67) = 2.52, *p* = .007, η_p_^2^ = .33, with ADFES, CK, BU-4DFE, STOIC performing best, followed by MMI, MPI, D3D-FACS, DISFA, DynEmo, GEMEP, DaFEx, UT Dallas, BINED, and finally FG-NET (see Fig. 1). The difference was statistically significant only between ADFES and FG-NET (*p* = .037). A significant main effect of emotion, *F*(5, 75) = 5.78, *p* < .001, η_p_^2^ = .28, further revealed that recognition rates were highest for happiness, followed by disgust, then surprise, sadness, and anger, and finally fear. Pairwise comparisons showed that happiness was better recognized than sadness (*p* = .009), anger (*p* = .003), fear (*p* < .001), and marginally better than surprise (*p* = .059). For none of the above analyses, the human vs. machine difference was significant (*F*s < 0.002, *p*s > .977), nor was there a significant interaction between database or emotion and human vs. machine (*F*s < 1.48, *p*s > .151).^3^

**Fig. 2:**
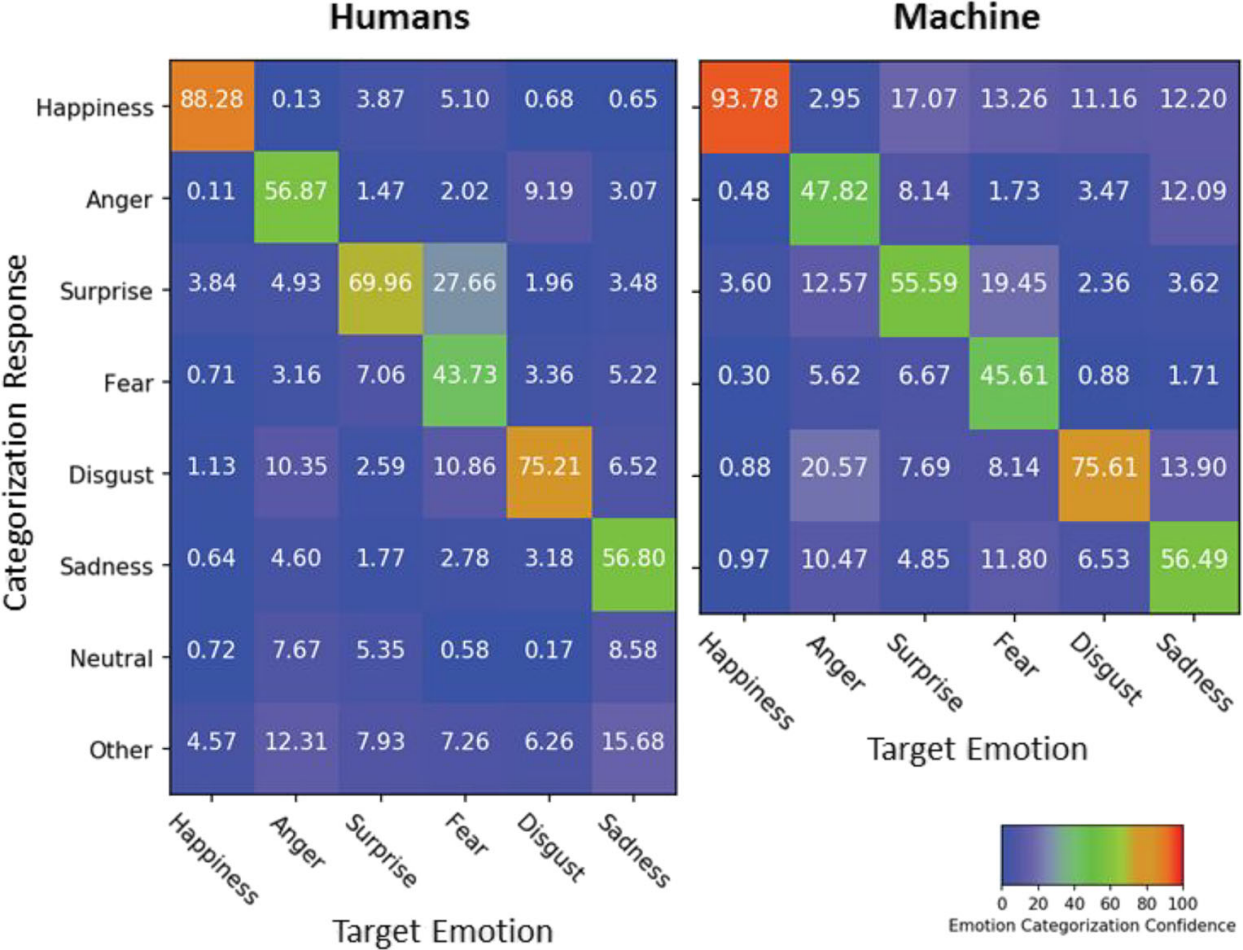
Confusion matrices of emotion categorization for human observers and the machine classifier averaged across database in Study 1

As shown in Fig. 2, confusion rates were generally below the 25% chance level, except for fear which was sometimes confused with surprise (27.66%) in humans. The same confusion arose in machine classification (19.45%). Also, there was a tendency for both humans and machine to label anger expressions as disgust (10.35% and 20.57%, respectively). In order to quantify the similarity of confusions between machine and human, each confusion matrix was transformed into a single vector (see Kuhn et al., 2017). Correlational analyses indicated a significant overlap between both matrices (*rho* = .71, *S* = 2256, *p* < .001), suggesting that recognition patterns of target and non-target emotions were positively related in humans and machine.

#### Intensity rating

**Fig. 3:**
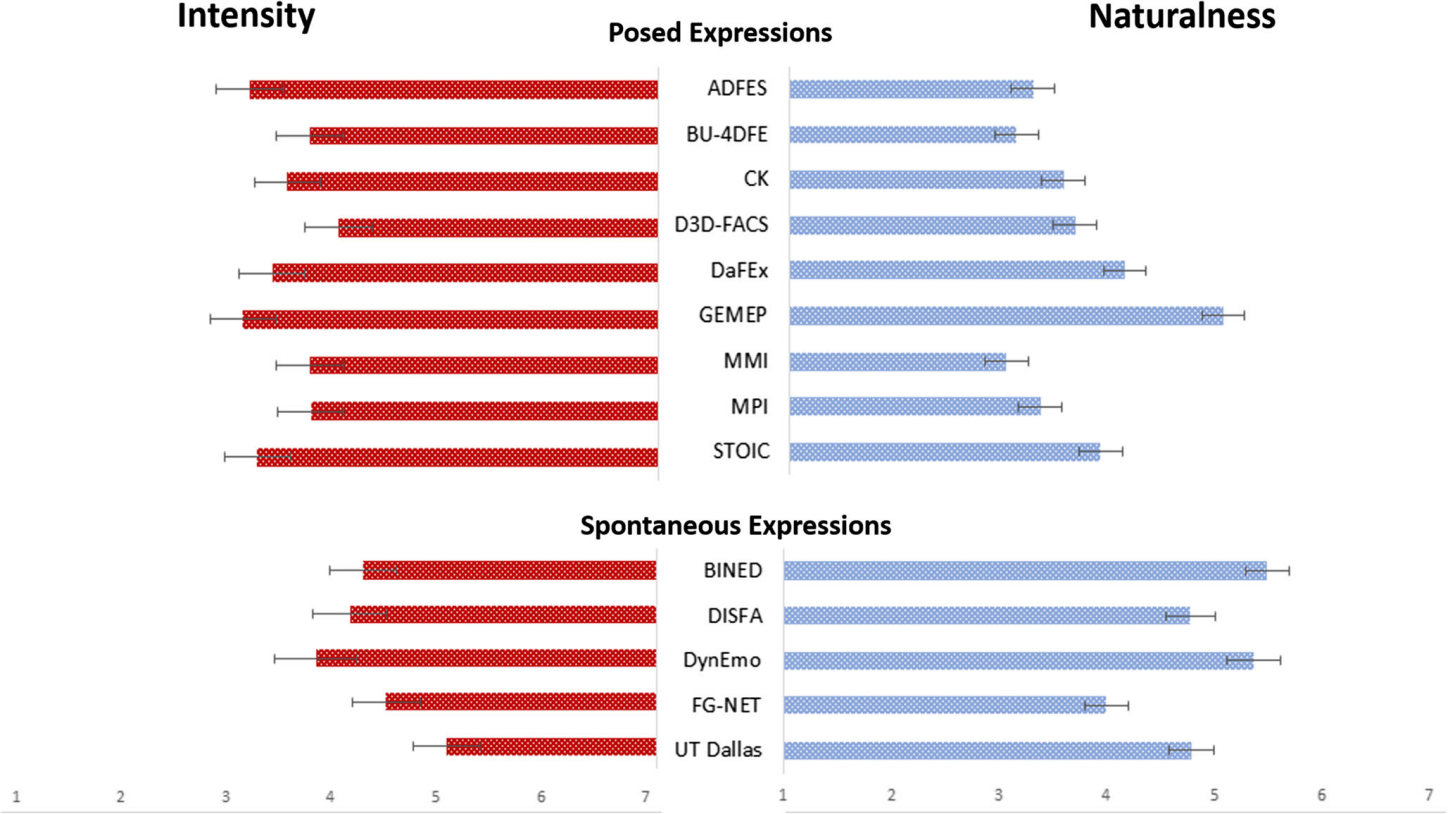
Mean intensity ratings (red bars) and naturalness ratings (blue bars) of human observers for posed and spontaneous expressions per database in Study 1. Error bars represent SEM

Results yielded a significant main effect of database, *F*(13, 67) = 2.89, *p* = .002, η_p_^2^ = .36, with GEMEP, ADFES, STOIC, and DaFEx attracting the highest scores in expression intensity, followed by CK, MMI, BU-4DFE, MPI, DynEmo, D3D-FACS, and finally DISFA, BINED, FG-Net, and UT Dallas (see Fig. 3). Pairwise comparisons showed that UT Dallas stimuli were rated as significantly less intense than those from GEMEP (*p* = .005), ADFES (*p* = .008), STOIC (*p* = .014), and DaFEx (*p* = .039). Overall, expressions from posed datasets (M = 4.53, SD = 0.76) were perceived as more intense than those from spontaneous sets (M = 3.66, SD = 0.85), *t*(79) = 4.64, *p* < .001, Cohen’s *d* = 1.07.

There was also a significant main effect of emotion, *F*(5, 75) = 2.87, *p* = .020, η_p_^2^ = .16, with disgust and fear being the two most intense expressions, followed by happiness, surprise, anger, and finally sadness. Pairwise comparisons showed that sadness was rated as significantly less intense than disgust (*p* = .009), and marginally significantly less intense than fear (*p* = .076). Overall, perceived intensity significantly predicted participants’ accuracy in emotion recognition, *β* = .50, *t*(79) = 5.07, *p* < .001, with better performance the more intense the expression was judged to be.

#### Naturalness rating

A significant main effect of database, *F*(13, 67) = 15.99, *p* < .001, η_p_^2^ = .76, revealed that BINED, DynEmo, GEMEP, UT Dallas, and DISFA achieved the highest scores in naturalness (*p*s = 1.0) (Fig. 3). Pairwise comparisons showed that BINED and DynEmo were rated as significantly more natural than DaFEx, FG-NET, STOIC, D3D-FACS, CK, MPI, ADFES, BU-4DFE, and MMI (*p*s < .05). GEMEP was rated as significantly more natural than FG-NET, STOIC, D3D-FACS, CK, MPI, ADFES, BU-4DFE, and MMI (*p*s < .05). UT Dallas and DISFA were rated as significantly more natural than D3D-FACS, CK, MPI, ADFES, BU-4DFE, and MMI (*p*s < .05), with DaFEx scoring significantly higher in naturalness than MMI (*p* = .024). In general, expressions from posed datasets (M = 3.69, SD = 0.76) were perceived to be less natural than those from spontaneous sets (M = 4.86, SD = 0.70), *t*(79) = – 6.66, *p* < .001, Cohen’s *d* = 1.59.

The main effect of emotion was not significant, *F*(5, 75) = 0.67, *p* = .644, η_p_^2^ = .04. Perceived naturalness significantly predicted participants’ accuracy in emotion recognition, *β* = – .28, *t*(79) = -2.62, *p* = .011, with worse performance the more natural the expression was judged to be.

### Discussion

The findings of the first study showed considerable variance in emotion recognition across the 14 databases ranging from 34% to 83%. On average, posed expressions were recognized better and judged as more intense (but less natural) than spontaneous ones. Intensity ratings in turn predicted recognition accuracy, with higher performance the more intense the expression. As such, posed stimuli may act as salient symbols of highly expressive and intense displays (Hess et al., 1997; Motley & Camden, 1988). Those can be easily identified, but are seen as less representative of everyday behavior (Barrett, 2011). When comparing human vs. machine performance there was strong convergence, yielding similar patterns of emotion classification and confusion between categories. This makes automated analysis a suitable tool for assessing facial expressions.

## Study 2

The second study intended to go beyond the limited subset of Study 1 and achieve a full validation of the 14 dynamic databases. For this, we processed the entire databases using automated methods for measuring emotion. We further analyzed the facial (AU) cues and technical features that may contribute to expression recognition.

### Method

In this study, we considered the entire stimulus array from each of the 14 databases comprising 5591 videos on the whole. Out of those, 1179 videos contained non-basic emotion labels (e.g., pride), yielding a total of 3812 videos of basic emotion expressions (1624 posed, 42.60%; 2188 spontaneous, 57.40%)^4^ from 855 encoders (536 females, 319 males) that were submitted to data analysis (see Table 1). In order to examine potential physical differences between the database stimuli, the following technical features were extracted using OpenFace 2.0 (Baltrusaitis et al., 2018) or FACET (see Table 3): video duration (mean, SD), face box size (mean, range) as the relative proportion of the visible facial area in a video frame, head rotation (up-down, left-right, head-tilt), and head motion (translational, rotational). As regards the last feature, we combined information from the individual movement parameters into one index to estimate rotational and translational head motion.

**Table 1.** Characteristics of the 14 databases

Database	Videos		Encoders		
	Basic Emotions	Total	Female	Male	Total
ADFES^1^	131	216	10	12	22
BINED^2^	492	492	45	37	82
BU-4DFE^3^	467	467	58	20	78
CK^4^	309	327	69	37	106
D3D-FACS^5^	67	463	6	4	10
DaFEx^6^	286	286	4	4	8
DISFA^7^	243	243	12	15	27
DynEmo^8^	151	358	82	69	151
FG-NET^9^	324	377	9	9	18
GEMEP^10^	50	145	5	5	10
MMI^11^	191	737	13	20	33
MPI^12^	80	439	4	4	8
STOIC^13^	60	80	5	5	10
UT Dallas^14^	961	961	214	78	292
TOTAL	3812	5591	536	319	855

Similar to Study 1, automated facial expression analysis was achieved by processing all video stimuli without baseline correction (cf., Stöckli et al., 2018). Where necessary, original videos were rotated into upright horizontal position and/or converted into Windows Media Video (.wmv) or MPEG-4 (.mp4) format to allow batch processing with FACET, while maintaining the original video resolution. Besides the classification of facial expressions in terms of the basic six emotions (anger disgust, fear, happiness, sadness, and surprise), we analyzed the machine data at the level of the individual facial actions: AU1, 2, 4, 5, 6, 7, 9, 10, 12, 14, 15, 17, 18, 20, 23, 24, 25, 26, 28, 43 (see Table 2 for AU definitions). We performed the same pre-processing steps and calculation of odds-based confidence scores for emotions/AUs as detailed in the first study.

**Table 2.** AU relative contribution to emotion recognition performance

Action Units		Emotion					
		Happiness	Surprise	Anger	Sadness	Disgust	Fear
AU1	Inner brow raiser	0.31	2.45	– 1.20	**13.60**	– 0.32	4.49
AU2	Outer brow raiser	1.13	**11.54**	– 0.34	– 3.13	– 0.06	0.63
AU4	Brow lowerer	– 1.52	0.06	**14.63**	2.61	0.13	– 0.13
AU5	Upper lid raiser	0.50	2.93	0.08	– 0.19	0.07	**7.06**
AU6	Cheek raiser	**10.77**	0.19	– 1.95	– 0.99	– 0.42	– 0.18
AU7	Lid tightener	0.13	– 0.73	**6.12**	0.17	3.02	– 0.88
AU9	Nose wrinkler	– 0.20	– 0.19	– 0.03	– 0.32	**18.99**	– 0.06
AU10	Upper lip raiser	– 1.19	– 0.55	– 0.95	– 1.02	**15.70**	0.07
AU12	Lip corner puller	**31.75**	0.12	0.49	– 0.58	– 1.94	– 0.10
AU14	Dimpler	**8.46**	– 0.53	– 2.12	0.62	– 0.42	– 0.19
AU15	Lip corner depressor	– 0.27	– 0.22	– 1.52	**10.87**	0.28	– 0.07
AU17	Chin raiser	– 0.12	– 0.14	2.75	4.72	0.72	0.04
AU18	Lip pucker	– 2.44	0.19	3.14	4.91	– 0.11	– 0.04
AU20	Lip stretcher	5.56	– 2.02	– 1.04	0.73	0.26	**6.96**
AU23	Lip tightener	0.04	– 0.16	**7.92**	– 1.45	0.03	– 0.05
AU24	Lip presser	– 0.87	0.47	4.02	0.44	0.00	0.06
AU25	Lips part	**13.87**	5.38	0.20	– 0.56	3.62	0.47
AU26	Jaw drop	– 3.35	**12.83**	– 0.38	0.03	– 0.81	– 0.51
AU28	Lips suck	1.51	0.03	2.11	0.43	– 0.08	0.01
AU43	Eye closure	1.71	2.36	2.04	2.58	3.06	0.43

*Note.* Regression coefficients (β) > 6.0 are printed in bold. The prior of p_0_ were happiness = 1, surprise = 3, anger = 4, sadness = 2, disgust = 1, fear = 5. See Table S2 for results per database

With reference to the criteria proposed in the Facial Action Coding (Ekman, Friesen, & Hager., 2002, p. 174; see also Table 4 in Krumhuber & Scherer, 2011), facial action (AU) configurations were further examined in association with specific basic emotions. For this, AU combinations indicative of full emotion prototypes or major variants thereof were scored as 1 or 0.75, respectively. Next, a weighted prototypicality score was computed by summing the FACET confidence scores of AUs within a combination, and multiplying the sum scores by 1 (full prototype) or 0.75 (major variant). This resulted in a total prototype score, with higher numbers reflecting greater emotional prototypicality.

### Results

The results yielded a large positive correlation between the machine performance on the small set (Study 1) and the big set (Study 2), *r*(81) = .65, *p* < .001, indicating that classification accuracy of the full databases could be predicted from the data of the small selective set.

#### Emotion recognition

The overall recognition accuracy of 55.51% (SD = 49.70) for the big set was significantly higher than chance (17%), *t*(3811) = 47.84, *p* < .001, Cohen’s *d* = .77, with all 14 databases passing the chance level threshold, *t*s > 2.40, *p*s < .02. In general, expressions from posed datasets (M = 70.32, SD = 45.70) were better recognized than those from spontaneous ones (M = 44.52, *SD* = 49.71), *t*(3641.32) = 16.60, *p* < .001, Cohen’s *d* = .54.

**Fig. 4:**
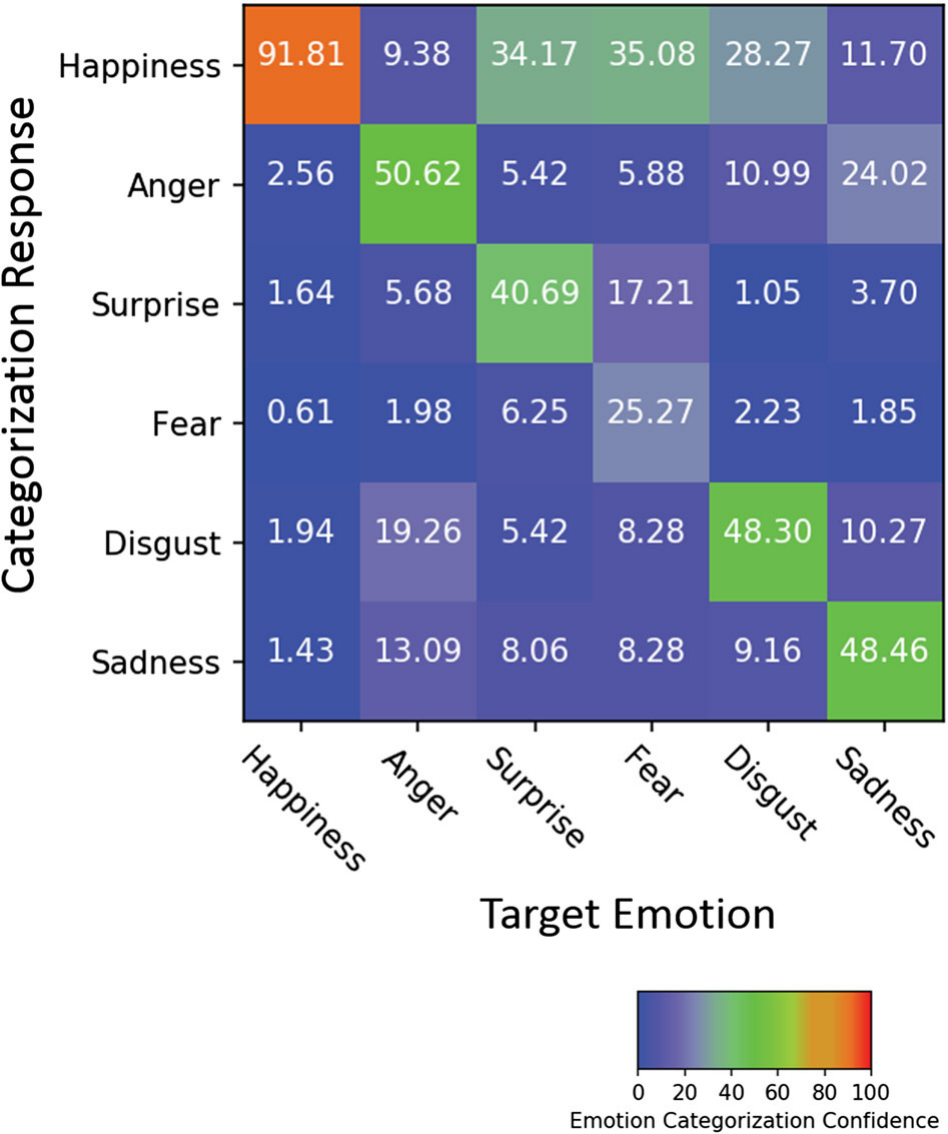
Emotion confusion matrix of the big set (*N* = 3812) averaged across database in Study 2. See Figure S1 for results per database

A 6 (emotion) x 14 (database) ANOVA showed a significant main effect of database, *F*(13, 3731) = 86.70, *p* < .001, η_p_^2^ = .23, with ADFES (M = 97%), CK (M = 97%), and STOIC (M = 80%) achieving the highest recognition scores, followed by BU-4DFE (M = 68%), MMI (M = 64%), UT Dallas (M = 58%), MPI (M = 55%), D3D-FACS (M = 54%), DaFEx (M = 52%), and FG-NET (M = 43%), and finally DISFA (M = 35%), GEMEP (M = 34%), BINED (M = 28%), and DynEmo (M = 26%). A main effect of emotion, *F*(5, 3731) = 87.99, *p* < .001, η_p_^2^ = .11, further revealed that happiness was recognized best, followed by anger, sadness, disgust, surprise, and finally fear (see Fig. 4). Pairwise comparisons with Games-Howell adjustment showed that happiness was better recognized, and fear was worse recognized than all other emotions (*p*s < .001). In addition, surprise was more poorly recognized than anger (*p* = .017) and disgust (*p* = .038).

**Fig. 5:**
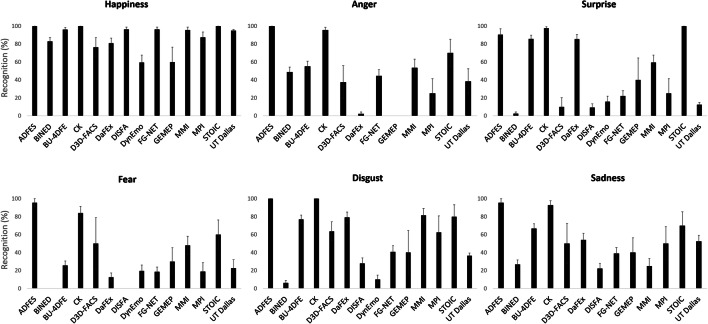
Mean recognition of the six basic emotions per database in Study 2. Error bars represent SEM. See Table S1 for multiple comparisons between the means for each emotion per database

In addition to the two main effects, the ANOVA revealed a significant interaction between emotion and database, *F*(62, 3731) = 11.85, *p* < .001, η_p_^2^ = .16. As shown in Fig. 5, cross-database classification performance was consistently high in the context of happiness, with recognition rates above 50%. However, there was considerable variance amongst the databases in the recognition of all other emotions. For anger and fear, the only datasets that achieved > 70% accuracy were ADFES and CK (and STOIC for anger), with markedly lower performance of the remaining datasets, i.e., DaFEx, GEMEP, and MPI. This result also applied to sets with spontaneous expressions such as BINED, DISFA, DynEmo, FG-NET and UT Dallas whose classification scores were amongst the lowest in the context of surprise, disgust, and sadness. BU-4DFE, DaFEx, and STOIC did reasonably well in conveying the latter three emotions, although their performance indices were not as high as those by ADFES and CK (see also Table S1 in the Supplementary Materials).

#### Confusion rates

When analyzing confusion rates in target emotion classification, a similar pattern occurred as in Study 1 (Fig. 4). Anger was likely to be confused with disgust (19.26%), whereas fear was often confused with surprise (17.21%). Furthermore, happiness was a commonly chosen label for emotions such as surprise, fear, and disgust, which might be due to the occurrence of smiling in those expressions.

**Fig. 6:**
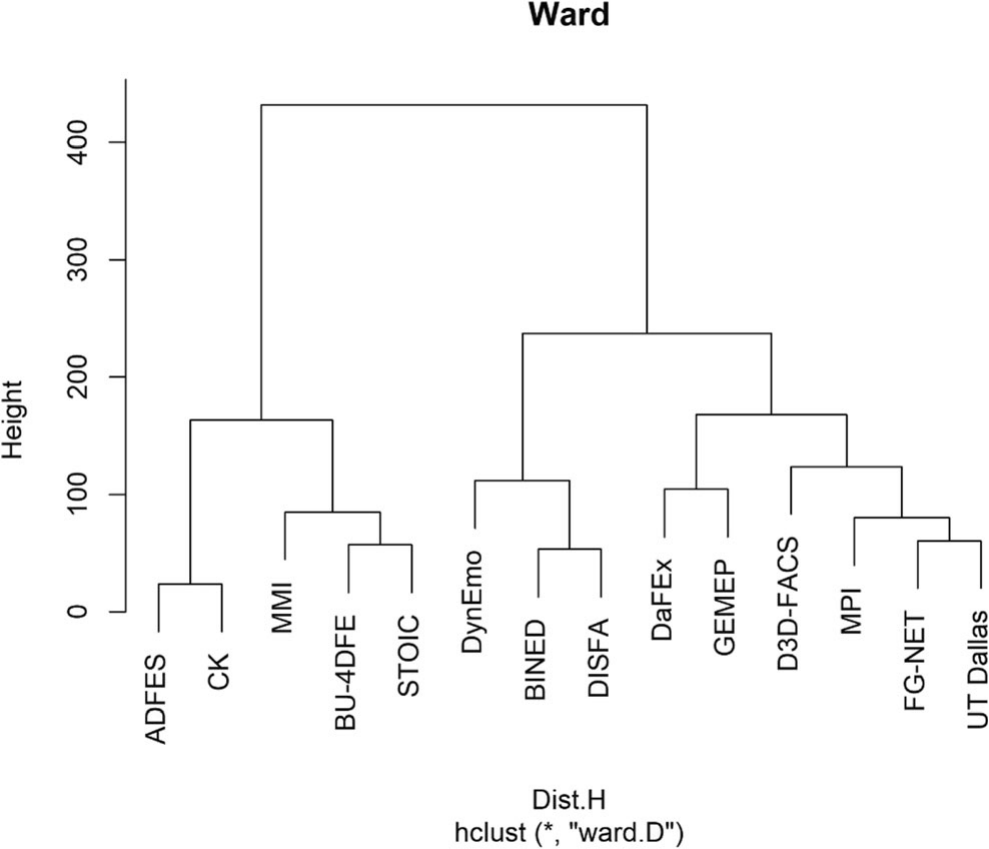
Dendrogram showing the hierarchical clustering of the 14 databases

In order to group databases by the similarity of their confusion patterns**,** a hierarchical cluster analysis was then performed. The average silhouette approach divided the 14 databases into two main clusters (Fig. 6). Cluster 1 was composed of ADFES, CK, MMI, BU-4DFE, and STOIC - the best performing databases with high overall accuracy scores. Cluster 2 comprised the remaining databases. ADFES and CK were further grouped into a sub-cluster that is characterized by accuracy rates > 83% for each predicted emotion and few confusion errors (see also Fig. S1). MMI, BU-4DFE, and STOIC made up the second sub-cluster with accuracy rates > 53% for happiness, anger, surprise, and disgust; however, anger was confused with disgust in more than 28% of all cases. With regard to DynEmo, BINED, and DISFA, individual accuracy scores were moderate (*<* 30%), except for happiness and anger (BINED), with surprise and fear being often confused with happiness (50–91%). The final sub-cluster consisted of DaFEx, GEMEP, D3D-FACS, MPI, FG-NET, and UT Dallas which is characterized by inconsistent and relatively frequent confusion errors.

#### Facial action units

Based on a detailed FACS analysis, we examined the extent to which the classification of the basic six emotions depends on individual facial actions. For this, the relative contribution of the 20 AUs to correct identification of the target emotion was calculated using Bayesian penalized regression analyses with a regularized horseshoe prior (Piironen, & Vehtari, 2017; Van Erp et al., 2019, the predicted number of non-zero coefficients was set to 1–5 according to the minimal number of prototype AUs for each emotion). Overall, happiness (R^2^ = 0.73) and disgust (R^2^ = 0.70) were the two best predicted emotions, followed by anger (R^2^ = 0.65), surprise (R^2^ = 0.50), sadness (R^2^ = 0.48), and finally fear (R^2^ = 0.38). When analyzing the results separately by the type of facial action, it can be seen that some AUs were more common than others (Table 2, see also Table S2). Specifically, the predictive power was highest for AUs that are hypothesized to signal a particular emotion according to Basic Emotion Theory (Ekman et al., 2002). These were AUs 6 (cheek raiser) and 12 (lip corner puller) for happiness, AUs 9 (nose wrinkler) and 10 (upper lip raiser) for disgust, AUs 4 (brow lowerer), 7 (lid tightener), and 23 (lip tightener) for anger, AUs 2 (outer brow raiser) and 26 (jaw drop) for surprise, AUs 1 (inner brow raiser) and 15 (lip corner depressor) for sadness, and AUs 5 (upper lid raiser) and 20 (lip stretcher) for fear.

An analysis of the emotion prototype scores further showed that expressions from posed datasets (M = 50.79, SD = 37.72) were more prototypical in their facial AU patterns than those from spontaneous ones (M = 34.32, SD = 34.81), *t*(3334.81) = 13.77, *p* < .001, Cohen’s *d* = .45 (see Figure S2 for results per database). A logistic regression analysis revealed that the prototypicality of an expression significantly predicted emotion recognition performance, standardized *β* = 1.05, *p* < .001, 95% CI [0.97, 1.13], thereby explaining 26.1% of the variance.

#### Technical features

Table 3 lists the technical features for each database, such as duration (mean, SD), face box size (mean, range), head rotation (up-down, left-right, head-tilt), and head motion (translational, rotational). As can be seen, there was considerable variability across databases. On average, video recordings from spontaneous databases seemed to be longer in duration, with a smaller visible area of the face and increased head rotation and motion. To test whether accuracy rates in emotion detection vary as a function of low-level visual properties of the stimuli, we conducted a multiple regression analysis with a random intercept estimate for database in R (3.6.1, R Core Team, 2016) using the lme4 package (Bates et al., 2015). From all technical features, the following four significantly predicted recognition performance: mean duration (*β* = 0.47, SE = 0.08, *z* = 5.78, *p* < .001), head-tilt (*β* = – 0.53, SE = 0.11, *z* = – 5.00, *p* < .001), translational motion (*β* = – 0.53, SE = 0.11, *z* = – 5.00, *p* < .001), and rotational motion (*β* = 0.18, SE = 0.08, *z* = 2.33, *p* < .02). The positive relationship between duration and accuracy suggests that slightly longer videos may be beneficial for classification. By contrast, head-tilt and translational motions appeared to negatively affect performance, whereas some variability in head rotation over time might be favorable by adding extra information (bold fonts in Table 3 indicate results per database).

**Table 3.** Technical features of the 14 databases

Database	Duration		Face Box Size		Head Rotation			Head Motion	
	*Mean*	*SD*	*Mean*	*Range*	Up-Down	Left-Right	Head-Tilt	Transla-tional	Rota-tional
ADFES	5.95	0.07	297.82	13.33	0.00	0.01	– 0.02	5.11	0.03
BINED	**43.14**	21.26	165.82	47.13	0.13	– 0.09	– 0.01	42.54	0.18
BU-4DFE	4.01	0.43	703.91	34.67	– 0.14	0.03	0.00	4.51	0.04
CK	0.69	0.35	243.55	9.02	0.00	0.02	– 0.01	5.33	0.03
D3D-FACS	4.15	1.16	494.07	85.76	0.00	0.57	– 0.03	3.50	0.05
DaFEx	10.64	3.34	110.39	9.88	– 0.06	0.04	– 0.02	9.72	0.07
DISFA	**26.90**	13.92	197.21	17.94	0.11	– 0.04	0.01	10.04	0.06
DynEmo	**98.89**	71.45	274.16	67.66	– 0.13	0.05	**0.00**	32.46	0.16
FG-NET	5.72	1.99	**210.18**	14.28	**0.02**	0.03	0.01	9.04	**0.06**
GEMEP	2.29	0.89	173.57	16.98	0.03	0.02	**– 0.01**	29.53	0.12
MMI	20.26	81.89	300.65	30.29	– 0.03	0.00	**– 0.01**	9.83	0.07
MPI	4.31	1.41	**119.58**	12.48	– 0.08	**0.02**	0.01	17.56	**0.12**
STOIC	0.52	0.00	198.90	11.30	– 0.02	0.02	– 0.01	0.74	0.02
UT Dallas	6.03	0.48	263.94	**15.78**	– 0.07	0.10	0.01	**5.67**	**0.04**

*Note*. Duration is given in seconds. Average face box size represents width by height in pixels as provided by FACET. Head motion was computed as the sum of the SDs of the “pose_T” (translational) and “pose_R” (rotational) estimates as provided by OpenFace. Bold font indicates significant predictors of emotion recognition performance

### Discussion

The full validation of the 14 databases again revealed considerable variance in recognition performance ranging from 26% to 97%. Similar to Study 1, posed stimuli were easier to identify in terms of their target emotion. This may be due to their saliency as idealized prototypes of affective displays as suggested in the literature (Barrett et al., 2019). By conducting a detailed FACS analysis, we could demonstrate that AU configurations indicative of basic emotions were indeed more common in posed stimuli. The prototypicality of an expression in turn predicted classification rates, with higher performance the more prototypical the facial behavior. Furthermore, accuracy varied with the technical features of each database, thereby pointing toward the modulating role of stimulus quality in expression recognition.

## General discussion

Based on a growing body of research arguing that facial displays of emotion are dynamic phenomena (Krumhuber et al., 2013; Sato et al., 2019), there has been a shift in interest towards dynamic expressions over the past two decades. This has led to a proliferation of stimuli available to the scientific community, amounting to a large number of datasets varying in size and properties (Krumhuber et al., 2017). While there are isolated attempts at validating dynamic stimulus sets, no cross-corpus evaluation exists to date that would allow for a robust comparison between the databases. The aim of the present research was to test different stimulus collections of dynamic facial expressions, thereby providing common validation data that can serve as a benchmark for future researchers.

In two studies, we showed that emotion classification accuracy considerably varied amongst the 14 databases. Overall, ADFES, CK and STOIC performed the best, achieving recognition rates over 80%. All three databases contain posed expressions produced upon instructions to perform a specific expression/facial action (Van der Schalk et al., 2011; Kanade et al., 2000; Roy et al., 2007). Such standardized tasks allow for clearly distinguishable displays that represent clear-cut exemplars of the emotion. In line with previous research (Motley & Camden, 1988; Calvo & Nummenmaa, 2016), observers endorsed the predicted emotion to a greater extent when behavior was posed than spontaneous. Deliberately posed displays were also perceived as more intense, with intensity ratings positively predicting participants’ level of recognition. Higher facial expressivity therefore seems to facilitate emotion decoding, implying an intrinsic link between expression intensity and recognition (Hess et al., 1997; Wingenbach et al., 2016).

From the set of posed databases, we recommend ADFES and CK for studies that aim for highly recognizable and intense expressions. Both demonstrate excellent recognition rates across the six emotion categories. CK also contains a particular large number of videos from a variety of different encoders which makes it a diverse stimulus set. While posed databases allow for strong emotional displays, these often reflect stereotypical and often exaggerated forms of behavior (Barrett, 2011). Such stylized patterns may not be representative of the facial actions seen in everyday life. In fact, emotions are typically expressed in subtle and varied ways (Fernández-Dols, 1999). Alternative choices may be DaFEx and GEMEP which comprise intense but less directed expressions. Although their recognition levels differed between the six emotion categories, they may be suitable for studies that focus on a subset of emotions. Both databases depict relatively few encoders (i.e., actors) who enact a range of emotion scenarios, and feature audiovisual portrayals.

In the present research, participants generally indicated lower levels of perceived naturalness for deliberately posed displays. Furthermore, machine analysis revealed more prototypical facial (AU) configurations when behavior was posed. Among the available set of spontaneous databases, we recommend DISFA and UT Dallas. They achieved moderately high ratings of naturalness, with recognition rates being in the acceptable range for some emotions, particularly in the case of UT Dallas. UT Dallas further contains large numbers of videos from different encoders, making it a rich set of spontaneous stimuli. At the technical level, it should be noted however that this database features parameters (e.g., face box range, head motion) that may affect emotion classification accuracy.

Together, the findings suggest that existing databases currently face a trade-off between realism and ecological validity on one end, and expression uniformity and comparability on the other. This could be problematic in the sense that the emotional content of posed recordings, both in terms of production and perception, does not translate to real-world settings. Until now, most human perception studies utilize highly recognizable portrayals of facial expressions. Moreover, automated methods mainly focus on prototypical expressions for training and testing (Pantic & Bartlett, 2007). In order to develop stimulus sets that mirror naturally occurring human affective behavior, it will be essential for future research to simulate real-world environments as closely as possible.

An important aspect in that regard relates to the technical setup in database construction. Posed expressions are typically captured under tightly restricted conditions, with near-frontal views, little head pose variation, and uniform background. While constant recording settings minimize potential differences in the low-level visual properties of the stimuli (Beringer et al., 2019; Calvo et al., 2018), such constrained input data are not normally found in spontaneous face databases. The present research showed that spontaneous expressions (despite being recorded in the laboratory) featured a smaller visible area of the face and more head rotation and motion. Spontaneous behavior therefore involves handling variability in stimulus settings which increases its complexity of recognition. This is particularly an issue for machine analysis, with many automated systems still being sensitive to the recording condition (Zeng et al., 2009). Here, we found that the technical features of each database significantly predicted performance rates. Unless posed and spontaneous portrayals satisfy the same methodological criteria, choices in corpus construction will indubitably induce perceptual confounds in emotion recognition.

To minimize trade-offs between expression realism and recognizability, researchers should move away from ideal laboratory conditions and directed facial action tasks in which expressions are produced in the exact same manner for each encoder. Face orientation and head poses are unlikely to be steady in daily life. Instead of a fixed recording position, it might be feasible to use head mounted cameras, thereby enabling encoders to move around more freely whilst keeping a constant viewing angle of the face. Such setup could be part of motion capture technologies that translate the movements of the person’s face into digitally constructed displays of emotion (Zhang, Snavely, N., Curless, B., & Seitz, 2004). Those have the advantage that certain features can be dealt with in a post-productive manner when building generative and/or morphable face models (e.g., Grewe, Le Roux, Pilz, & Zachow, 2018), thereby providing fine-grained control over the type and dynamics of facial actions that drive response classification. Generative approaches such as the one pursued by Yu, Garrodd, and Schyns (2012) also allow for facial models that are constructed based on ecologically valid facial movements, with the liberty to synthesize arbitrary facial expressions from parameterized movements.

While some of the existing databases contain high-resolution 3D scans for facial analysis and synthesis (e.g., BU-4DFE, D3D-FACS, MPI), smaller face sizes of emotion-evoked expressions highlight potential issues with stimulus quality. At the moment, spontaneous databases often lag behind posed ones in providing top-notch, technically sound, materials (Sandbach et al., 2012). To ensure high recording quality, a distinction could be made between what the camera sees and the setting in which the behavior occurs. To this end, a natural environment could be created that keeps sufficient spontaneity, while at the same time the visible area that is captured by the camera remains tightly controlled. Alternatively, a minimal context may be defined that describes the specific situation in which the recording is made (Bänziger & Scherer, 2007). Considerable research suggests that emotions are strongly context-dependent (Greenaway et al., 2018). Also, situational context determines the emotional meaning and significance of facial expressions (Maringer et al., 2011; Aviezer et al., 2017). For maximizing both the natural aspects of expression and recognition, integrating contextual information could thus help specify the emotional content of the recordings; an approach that mirrors human perception but also benefits automated methods which traditionally have been context insensitive (Calvo & D’Mello, 2010). For this, advanced annotations in the form of well-labeled data are a necessary prerequisite. To date, most databases still lack metadata about the emotion-eliciting context (i.e., utilized stimuli, environment, presence of other people, etc.). Failure to do so may contribute to the difficulty of recognizing emotions, particularly from spontaneous expressions.

In line with previous research, responses were more accurate for happy expressions, acting as the only positive emotion in this study. By contrast, recognition rates were lowest for fear which was often confused with surprise (Calvo & Nummenmaa, 2016), thereby sharing similar patterns of facial actions (Ekman et al., 2002). While database performance was consistently high in the context of happiness, there was considerable variance for all other emotions. As such, it seems that different databases are more or less suitable for portraying specific emotions. Following traditional approaches, we targeted the basic six emotions as the most commonly used categories for stimulus collection. The view that underlies this notion is rooted in theoretical assumptions that conceptualize emotions as discrete and fundamentally different. According to Basic Emotion Theory, a small number of categorical emotions exists that are basic or primary in the sense that they form the core emotional repertoire (Ekman, 1992; Ekman & Cordaro, 2011). While the discrete perspective remains influential (Cordaro et al., 2018), the narrow focus on a few, supposedly fundamental, emotions has increasingly been criticized (Barrett et al., 2019; Kappas et al., 2013).

Also, there is debate about whether facial expressions are necessarily linked to emotions or other affective, motivational, or socio-cultural factors (Fernández-Dols & Russell, 2017). Here, we focused on expressions produced in the laboratory. In real life, posed displays may occur in interpersonal contexts for a variety of reasons (e.g., to be polite, prevent conflicts, or strategically mask one’s true feelings), with spontaneous expressions being subject to the influence of multiple factors outside the emotion-eliciting situation (e.g., social presence of other people). Also, facial expressions typically fulfil a variety of functions (e.g., cognitive appraisals, action tendencies, social motives) and encompass a blend of affective and/or cognitive processes (Kappas et al., 2013; Parkinson, 2005) which may affect, alone or in combination, their recognizability.

To address some of these criticisms, a few promising efforts have lately aimed to extend the range of emotions and include non-basic affective states. Some of the databases examined here (i.e., DynEmo, GEMEP, MPI) reflect that approach by providing a wider array of affective displays such as those expressing embarrassment, boredom, or admiration. Furthermore, there are tentative efforts to detect basic and compound emotions “in the wild”, featuring a wide range of natural expressions (Benitez-Quiroz, Srinivasan, & Martinez, 2016). It falls to future research to review and validate stimulus collections that go beyond the basic emotion perspective. This may be informative not only for theory advancement but highlight potential applications in research using posed vs. spontaneous expressions. The present work constitutes a first step in providing cross-corpus validation data for 14 databases of dynamic facial expressions. We hope that this proves useful as a benchmark for accelerating future progress in the field.

## Electronic supplementary material

ESM 1 (DOCX 615 kb)

## Footnotes

1 Portrayals labelled as amusement (BINED, DynEmo) or joy (ADFES, DISFA, GEMEP) were included under the umbrella of happiness. Missing portrayals of surprise were substituted in one database (MPI) with those of disbelief, which belongs to the same emotion family (Shaver et al., 1987). Since D3DFACS does not include any emotion labels, we opted for stimuli with Action Unit configurations that are characteristic of the six basic emotions as proposed in the Facial Action Coding System manual (FACS, Ekman et al., 2002).

2 The presentation format slightly differed between participants, such that some participants (*N* = 70) saw a fixed set of 81 portrayals, whereas others (*N* = 54) were presented with a randomly selected set of 81 out of the 162 portrayals. Recognition rates between the two presentation formats were highly correlated, *r*(81) = .910, *p* < .001. Also, there was no significant difference in recognition accuracy between the two portrayals of an emotion, *t*(160) = – 1.16, *p* = .249, *d* = .09. We therefore collapsed them into one data file and averaged across the two portrayals per emotion and database

3 Given the potential risk for emotion portrayals with neutral expressive elements to be incorrectly classified as neutral by the machine, the neutral response category was left out. As no videos with intended neutral expression were analyzed, the exclusion of this response category does not impair the dominant emotion categorization (see Frank & Stennett, 2001; Skiendziel et al., 2019). In order to account for the unequal number of response categories in humans vs. machine, we ran additional analyses in which we post-experimentally removed the ‘neutral’ and ‘other’ answer options from the human data. The pattern of results remained the same. Recognition accuracy in humans was higher for posed than spontaneous expressions, *t*(37.82) = 3.20, *p* = .003, *d* = .36. In ANOVAs with the factors database (14) or emotion (6), the main effect of database, *F*(13, 67) = 2.31, *p* = .013, η_p_^2^ = .31, as well as emotion, *F*(5, 75) = 6.02, *p* < .001, η_p_^2^ = .29, remained significant. For none of the above analyses, the human vs. machine difference was significant (*F*s < 2.44, *p*s > .123), nor was there a significant interaction between database or emotion and human vs. machine (*F*s < 1.59, *p*s > .113).

4 The whole sets of MMI and CK (plus its extension CK+) contain spontaneous as well as posed expressions. While portrayals of both types were analyzed from the MMI database (174 posed, 17 spontaneous), it was impossible for the dataset authors of CK to identify the relevant spontaneous expressions (*N* = 122) within their set (personal communication). We therefore treated all CK portrayals as posed expressions
