# Supplementary material for: Human and machine validation of 14 databases of dynamic facial expressions
Source: Behav Res Methods. 2020 Aug 17;53(2):686–701. doi: 10.3758/s13428-020-01443-y (PMC8062366; doi:10.3758/s13428-020-01443-y)
Supplement: Supplementary file 1 — (DOCX 615 kb) [file 13428_2020_1443_MOESM1_ESM.docx]

**Supplementary Materials**

Human and Machine Validation of 14 Databases of Dynamic Facial Expressions

By E. G. Krumhuber, D. Küster, S. Namba, & L. Skora

*Table S1*. Mean classification accuracy and standard deviations for each emotion per database in Study 2.

|  |  | **Emotion** | | | | | | |  |
| --- | --- | --- | --- | --- | --- | --- | --- | --- | --- |
| **Database** |  | **Anger** | **Disgust** | **Fear** | **Happiness** | **Sadness** | **Surprise** | **Overall** | |
| **ADFES** | ***Mean*** | 100.00*_a_* | 100.00*_a_* | 95.45*_a_* | 100.00*_a_* | 95.45*_a_* | 90.48*_a_* | 96.95 | |
|  | ***SD*** | 0.00 | 0.00 | 21.32 | 0.00 | 21.32 | 30.08 | 17.27 | |
|  | ***N*** | 22 | 22 | 22 | 22 | 22 | 21 | 131 | |
| **BINED** | ***Mean*** | 48.78*_c_* | 6.10*_a_* | 0.00*_a_* | 82.93*_b_* | 26.83*_d_* | 2.44*_a_* | 27.85 | |
|  | ***SD*** | 50.29 | 24.08 | 0.00 | 37.86 | 44.58 | 15.52 | 44.87 | |
|  | ***N*** | 82 | 82 | 82 | 82 | 82 | 82 | 492 | |
| **BU-4DFE** | ***Mean*** | 55.13*_c_* | 76.92*_bd_* | 25.64*_f_* | 96.15*_e_* | 66.67*_abc_* | 85.71*_ade_* | 67.67 | |
|  | ***SD*** | 50.06 | 42.41 | 43.95 | 19.36 | 47.45 | 35.22 | 46.83 | |
|  | ***N*** | 78 | 78 | 78 | 78 | 78 | 77 | 467 | |
| **CK** | ***Mean*** | 95.56*_a_* | 100.00*_a_* | 84.00*_a_* | 100.00*_a_* | 92.86*_a_* | 97.59*_a_* | 96.76 | |
|  | ***SD*** | 20.84 | 0.00 | 37.42 | 0.00 | 26.23 | 15.43 | 17.72 | |
|  | ***N*** | 45 | 59 | 25 | 69 | 28 | 83 | 309 | |
| **D3D-FACS** | ***Mean*** | 37.50*_ab_* | 63.64*_a_* | 50.00*_ab_* | 76.47*_a_* | 50.00*_ab_* | 10.00*_b_* | 53.73 | |
|  | ***SD*** | 51.75 | 49.24 | 57.74 | 43.72 | 54.77 | 31.62 | 50.24 | |
|  | ***N*** | 8 | 22 | 4 | 17 | 6 | 10 | 67 | |
| **DaFEx** | ***Mean*** | 2.13*_c_* | 79.17*_ab_* | 12.50*_c_* | 80.85*_ab_* | 54.17*_a_* | 85.42*_b_* | 52.45 | |
|  | ***SD*** | 14.59 | 41.04 | 33.42 | 39.77 | 50.35 | 35.67 | 50.03 | |
|  | ***N*** | 47 | 48 | 48 | 47 | 48 | 48 | 286 | |
| **DISFA** | ***Mean*** | - | 27.78*_a_* | 0.00*_b_* | 96.30*_c_* | 22.22*_a_* | 9.26*_ab_* | 34.57 | |
|  | ***SD*** | - | 45.21 | 0.00 | 19.06 | 41.96 | 29.26 | 47.66 | |
|  | ***N*** | - | 54 | 27 | 54 | 54 | 54 | 243 | |
| **DynEmo** | ***Mean*** | - | 10.00*_b_* | 19.44*_b_* | 59.46*_a_* | - | 15.79*_b_* | 25.83 | |
|  | ***SD*** | - | 30.38 | 40.14 | 49.77 | - | 36.95 | 43.91 | |
|  | ***N*** | - | 40 | 36 | 37 | - | 38 | 151 | |
| **FG-NET** | ***Mean*** | 44.44*_c_* | 40.74*_bc_* | 18.52*_b_* | 96.30*_a_* | 38.89*_bc_* | 22.22*_bc_* | 43.52 | |
|  | ***SD*** | 50.16 | 49.60 | 39.21 | 19.06 | 49.21 | 41.96 | 49.65 | |
|  | ***N*** | 54 | 54 | 54 | 54 | 54 | 54 | 324 | |
| **GEMEP** | ***Mean*** | 0.00*_b_* | 40.00*_ab_* | 30.00*_ab_* | 60.00*_a_* | 40.00*_ab_* | 40.00*_ab_* | 34.00 | |
|  | ***SD*** | 0.00 | 54.77 | 48.30 | 51.64 | 51.64 | 54.77 | 47.85 | |
|  | ***N*** | 10 | 5 | 10 | 10 | 10 | 5 | 50 | |
| **MMI** | ***Mean*** | 53.57*_cd_* | 81.48*_ad_* | 48.00*_bcd_* | 95.65*_a_* | 25.00*_c_* | 59.46*_bd_* | 63.87 | |
|  | ***SD*** | 50.79 | 39.58 | 50.99 | 20.62 | 44.10 | 49.77 | 48.16 | |
|  | ***N*** | 28 | 27 | 25 | 46 | 28 | 37 | 191 | |
| **MPI** | ***Mean*** | 25.00*_b_* | 62.50*_ab_* | 18.75*_b_* | 87.50*_a_* | 50.00*_ab_* | 25.00*_b_* | 55.00 | |
|  | ***SD*** | 46.29 | 51.75 | 40.31 | 33.60 | 53.45 | 46.29 | 50.06 | |
|  | ***N*** | 8 | 8 | 16 | 32 | 8 | 8 | 80 | |
| **STOIC** | ***Mean*** | 70.00*_a_* | 80.00*_a_* | 60.00*_a_* | 100.00*_a_* | 70.00*_a_* | 100.00*_a_* | 80.00 | |
|  | ***SD*** | 48.30 | 42.16 | 51.64 | 0.00 | 48.30 | 0.00 | 40.34 | |
|  | ***N*** | 10 | 10 | 10 | 10 | 10 | 10 | 60 | |
| **UT Dallas** | ***Mean*** | 38.46*_b_* | 36.47*_bd_* | 22.72*_b_* | 94.99*_a_* | 52.54*_b_* | 12.44*_c_* | 57.86 | |
|  | ***SD*** | 50.64 | 48.23 | 42.89 | 21.85 | 50.36 | 33.08 | 49.40 | |
|  | ***N*** | 13 | 255 | 22 | 419 | 59 | 193 | 961 | |
| **Overall** | ***Mean*** | 50.62 | 48.30 | 25.27 | 91.81 | 48.46 | 40.69 | 55.51 | |
|  | ***SD*** | 50.06 | 50.00 | 43.50 | 27.43 | 50.03 | 49.16 | 49.70 | |
|  | ***N*** | 405 | 764 | 459 | 977 | 487 | 720 | 3812 | |
| *Note.* Row means not sharing a common subscript differ significantly at *p* < .05 (Games-Howell adjustment for multiple comparisons). | | | | | | | | | |


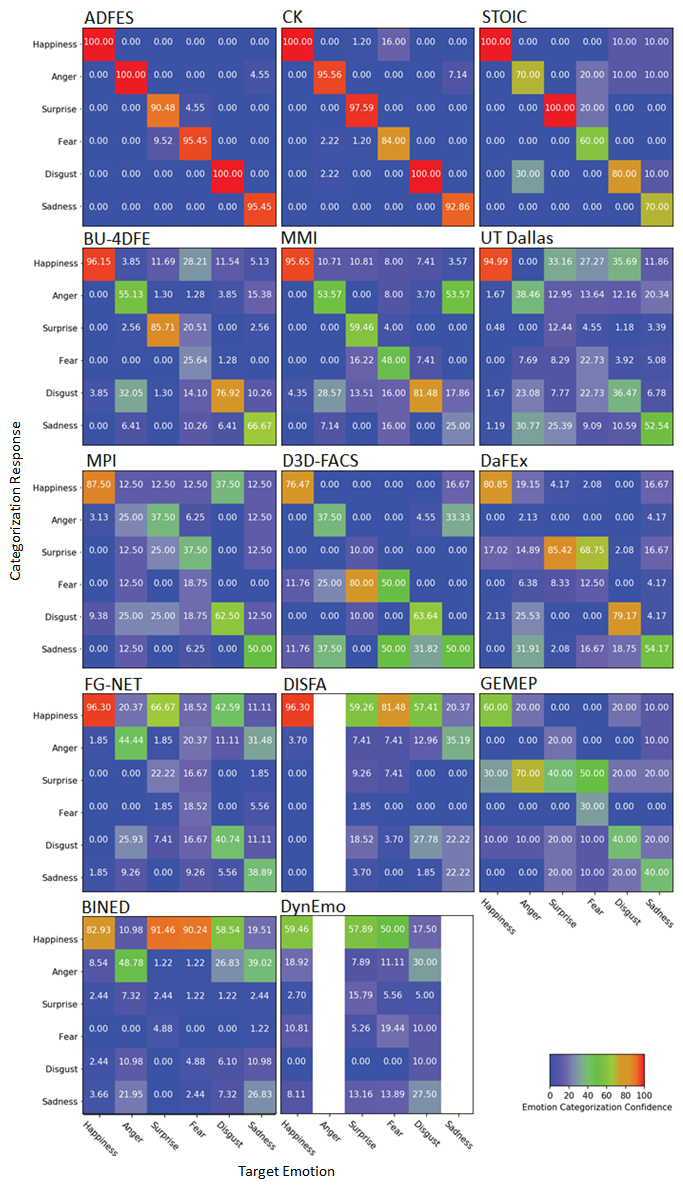


*Figure S1.* Confusion matrices of emotion categorization per database in Study 2.


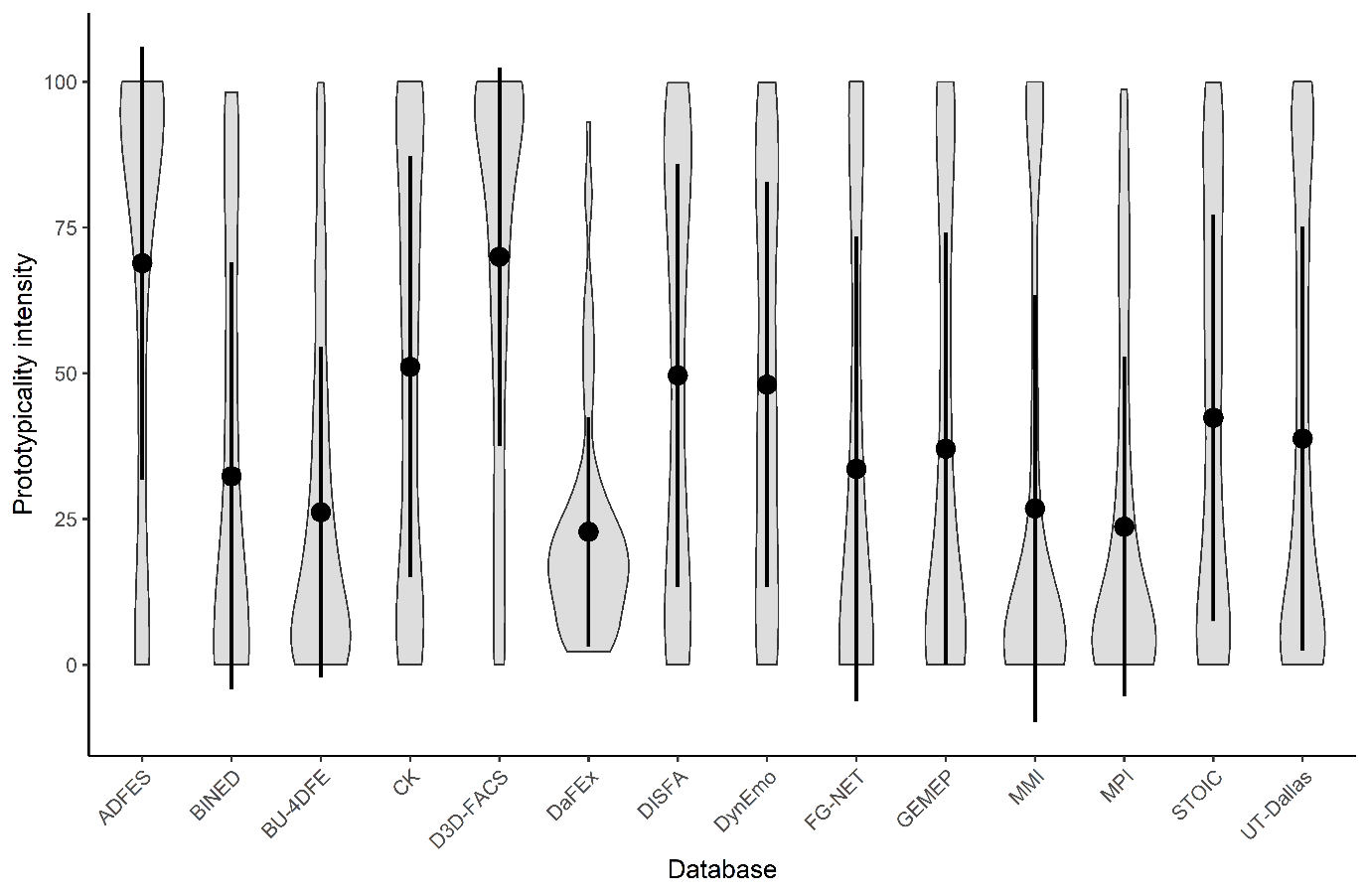


*Figure S2*. Degree of facial emotion prototypicality displayed by each database (dot = mean, vertical line = + 1SD, violin = frequency of occurrence).

| *Table S2*. AU relative contribution to emotion recognition performance by database. | | | | | | | | | | | | | | | | | | | | |  |
| --- | --- | --- | --- | --- | --- | --- | --- | --- | --- | --- | --- | --- | --- | --- | --- | --- | --- | --- | --- | --- | --- |
|  | **Action Unit** | | | | | | | | | | | | | | | | | | | |  |
| Database | 1 | | 2 | 4 | 5 | 6 | 7 | 9 | 10 | 12 | 14 | 15 | 17 | 18 | 20 | 23 | 24 | 25 | 26 | 28 | 43 |
| **anger** |  | |  |  |  |  |  |  |  |  |  |  |  |  |  |  |  |  |  |  |  |
| ADFES | -0.51 | | 0.14 | **13.53** | 0.05 | 0.03 | 0.59 | 0.04 | 0.02 | 0.07 | 1.09 | -0.54 | 0.22 | 0.67 | 0.02 | **12.99** | **12.92** | 0.03 | 0.04 | 0.39 | 0.09 |
| BINED | -1.82 | | -1.18 | **17.40** | **6.22** | -0.85 | **8.28** | 2.13 | -0.48 | -1.29 | -2.45 | -7.21 | 4.08 | 2.69 | -1.33 | **7.93** | 3.30 | -0.18 | -2.88 | 1.11 | -0.36 |
| BU-4DFE | -0.57 | | -0.33 | **10.09** | -0.29 | -3.81 | **9.19** | 0.62 | -0.93 | -0.03 | -7.60 | -1.09 | 1.99 | 3.03 | -0.38 | **8.70** | **9.81** | -0.54 | -0.10 | 2.22 | 3.67 |
| CK | -1.78 | | 0.22 | **14.06** | 0.12 | -0.26 | **6.94** | 0.03 | -0.78 | 0.13 | -2.13 | -2.39 | 3.60 | 1.90 | -0.21 | **6.93** | **10.77** | 0.08 | 0.02 | 1.48 | -8.10 |
| D3D-FACS | -0.95 | | -1.90 | 2.45 | 2.43 | -0.90 | 1.04 | 0.22 | -0.62 | -1.04 | -3.67 | -1.50 | 2.77 | 0.80 | -0.50 | 2.84 | 5.45 | 0.01 | -0.02 | -0.59 | 0.94 |
| DaFEx | -0.06 | | 0.01 | **18.33** | 0.00 | -0.25 | 1.22 | 0.04 | -0.02 | 0.05 | -7.77 | -1.38 | 5.75 | -0.23 | -0.39 | 5.28 | 0.02 | 0.05 | 0.00 | **11.86** | 0.09 |
| DISFA | 0.24 | | -0.97 | **17.46** | -3.28 | -0.79 | 1.49 | -0.12 | -1.80 | 0.21 | -0.20 | -8.50 | **6.36** | 4.50 | -1.19 | **8.63** | -2.31 | 0.03 | 0.48 | -0.48 | 0.06 |
| DynEmo | -0.77 | | -0.14 | **13.08** | 0.64 | -0.49 | 1.95 | 0.14 | -0.55 | -0.78 | -5.12 | -0.71 | -0.13 | 0.01 | -0.80 | 0.55 | **11.27** | -0.46 | -0.12 | 1.93 | 4.81 |
| FG-NET | -3.13 | | -0.31 | **15.55** | 0.11 | -3.00 | **6.29** | -0.78 | -2.13 | 0.52 | 3.12 | -1.57 | 2.55 | 4.93 | -2.28 | 5.61 | **6.37** | 0.08 | -1.15 | 4.76 | -2.97 |
| GEMEP | 0.17 | | -0.13 | -11.46 | 0.33 | -0.40 | **19.10** | -1.91 | 0.79 | 0.00 | **34.47** | -20.37 | -12.23 | 1.33 | -0.61 | 0.01 | **32.94** | 0.16 | 0.05 | 1.57 | -0.52 |
| MMI | -0.46 | | -0.47 | **16.96** | 0.00 | -1.22 | **8.50** | 0.10 | -0.01 | -0.35 | 0.09 | 0.29 | 0.55 | 4.62 | -0.90 | **17.95** | 0.80 | -0.13 | 0.14 | -0.61 | 5.11 |
| MPI | -0.81 | | 0.39 | **29.19** | 0.20 | -0.44 | 3.00 | -0.50 | -3.99 | -0.09 | -15.58 | -0.47 | **7.22** | 2.05 | 0.08 | **8.06** | **14.34** | -0.12 | -0.07 | -1.38 | 0.73 |
| STOIC | -1.57 | | -0.26 | **17.15** | -0.27 | -1.83 | 1.80 | -0.26 | -0.25 | -0.44 | -0.30 | -0.15 | -0.17 | 2.19 | 0.06 | **6.53** | 1.48 | 0.53 | -0.32 | 0.01 | -0.06 |
| UT Dallas | -2.10 | | 0.36 | **12.10** | 0.39 | -1.45 | 2.65 | -0.14 | -0.99 | -0.46 | -2.20 | -0.31 | 1.28 | 2.95 | -1.06 | **6.34** | 3.33 | -0.07 | -0.19 | 0.76 | 2.32 |
| **disgust** |  | |  |  |  |  |  |  |  |  |  |  |  |  |  |  |  |  |  |  |  |
| ADFES | -0.01 | | -0.02 | 0.06 | -0.02 | -0.02 | **9.65** | **18.96** | **12.67** | -0.04 | -0.46 | -0.10 | -0.20 | -0.54 | 0.10 | -0.50 | -0.25 | 0.11 | -0.02 | -0.11 | -0.05 |
| BINED | 0.00 | | 0.15 | 0.04 | 3.44 | -0.68 | -0.07 | **13.32** | **21.55** | -0.65 | -0.06 | 0.84 | 2.45 | 0.00 | -0.13 | 0.44 | -0.08 | -0.01 | 0.17 | 0.04 | 1.89 |
| BU-4DFE | -0.87 | | -0.06 | 2.23 | 0.00 | -0.71 | 0.60 | **17.27** | **17.44** | -3.31 | -0.06 | -0.01 | 0.06 | -0.36 | 0.36 | -0.69 | -0.35 | 5.94 | -0.68 | -0.11 | 2.10 |
| CK | -0.01 | | 0.00 | -0.04 | -0.01 | 0.02 | 0.08 | **19.36** | **13.05** | -0.01 | -0.01 | 0.01 | -0.01 | -0.07 | 0.10 | -0.04 | -0.03 | 0.02 | -0.01 | 0.00 | 0.74 |
| D3D-FACS | -1.75 | | -1.57 | -3.19 | -2.25 | 4.62 | 0.15 | 2.18 | **16.60** | -1.80 | -2.14 | 0.11 | 1.86 | 0.08 | -0.56 | 0.91 | -1.57 | -0.11 | 0.04 | -1.71 | 4.23 |
| DaFEx | -2.44 | | -1.28 | -0.07 | -1.44 | -3.18 | 3.83 | **15.71** | **12.71** | -6.43 | -2.64 | 1.87 | 0.62 | -0.99 | 0.10 | 0.58 | -3.91 | **8.49** | -8.15 | -0.31 | 1.14 |
| DISFA | -0.98 | | 0.05 | -0.61 | -1.41 | -0.50 | 13.14 | **15.75** | **16.42** | -2.09 | -0.37 | -0.82 | 1.84 | -0.57 | -2.73 | 0.11 | 0.00 | **8.23** | 0.41 | -0.04 | **8.03** |
| DynEmo | -0.14 | | -0.07 | -0.14 | -0.11 | -0.23 | 1.31 | **14.82** | **6.65** | -0.36 | -0.23 | -0.05 | -0.03 | 0.07 | 0.10 | -0.07 | -0.11 | 0.50 | -0.07 | -0.06 | 0.22 |
| FG-NET | 1.20 | | -0.50 | -0.42 | -0.93 | 0.46 | 3.47 | **19.85** | **16.72** | -1.54 | -0.27 | -0.61 | 0.07 | -2.04 | -2.23 | 0.74 | 0.06 | 0.91 | -0.10 | -0.06 | 0.45 |
| GEMEP | -0.65 | | -0.38 | 0.11 | -0.02 | -0.08 | 0.21 | 0.01 | 0.10 | -0.14 | 0.06 | 0.18 | 0.05 | 0.18 | 1.03 | 0.06 | 0.04 | 0.24 | -0.02 | 0.10 | 0.03 |
| *Table S2.* (Continued) | | | | | | | | | | | | | | | | | | | | | |
|  | | **Action Unit** | | | | | | | | | | | | | | | | | | | |
| Database | | 1 | 2 | 4 | 5 | 6 | 7 | 9 | 10 | 12 | 14 | 15 | 17 | 18 | 20 | 23 | 24 | 25 | 26 | 28 | 43 |
| MMI | | -1.46 | -1.29 | -0.75 | 0.17 | -0.88 | 0.08 | **16.24** | **13.84** | -3.20 | -0.52 | 0.29 | 5.09 | -0.95 | -0.65 | -0.95 | -1.67 | 2.33 | -2.82 | -0.42 | 1.79 |
| MPI | | 0.32 | 0.08 | -2.90 | -0.27 | -0.88 | 3.22 | **18.82** | **33.72** | -3.29 | -0.71 | 4.37 | -1.03 | -0.27 | -0.27 | -0.46 | -0.36 | 0.56 | -0.41 | 1.58 | 0.96 |
| STOIC | | -0.08 | -0.24 | -0.33 | -0.07 | -0.12 | 0.11 | **18.08** | **13.37** | -0.23 | -0.14 | -0.38 | -0.01 | -0.06 | -0.13 | -0.15 | -0.09 | 0.61 | -0.20 | -0.05 | -0.09 |
| UT Dallas | | -0.42 | 0.28 | 0.71 | 0.18 | -1.55 | 4.51 | **19.60** | **15.92** | -1.11 | -0.32 | 0.50 | 0.40 | -0.06 | -0.49 | -1.03 | -0.48 | 4.63 | -0.70 | -0.28 | 4.91 |
| **fear** | |  |  |  |  |  |  |  |  |  |  |  |  |  |  |  |  |  |  |  |  |
| ADFES | | **9.64** | 0.87 | -0.67 | **11.20** | -0.29 | -0.20 | -0.26 | -0.23 | -0.42 | -0.11 | -0.54 | -0.15 | -0.20 | 4.40 | -0.20 | -0.42 | -0.09 | -0.40 | 0.05 | -0.15 |
| BINED | | 0.12 | 1.17 | 0.02 | 1.37 | -0.06 | -0.14 | -0.02 | -0.01 | -0.18 | 0.14 | 0.11 | 0.09 | -0.06 | 5.92 | 0.00 | -0.02 | -0.08 | 0.05 | 0.02 | 0.44 |
| BU-4DFE | | 3.56 | -0.08 | -0.02 | 2.27 | -0.09 | -0.13 | 0.07 | -0.04 | 0.03 | -0.15 | -0.07 | 0.02 | -0.11 | 4.91 | -0.01 | 0.02 | -0.06 | -0.13 | 0.01 | -0.09 |
| CK | | **9.18** | -2.34 | -0.09 | **7.15** | 0.06 | 0.03 | 0.02 | 0.19 | 0.02 | -0.05 | -0.08 | -0.06 | -0.04 | **11.99** | 0.01 | 0.00 | 0.03 | -0.05 | 0.06 | -0.04 |
| D3D-FACS | | -0.62 | **16.38** | -0.13 | **7.89** | -3.20 | -0.35 | -0.70 | -0.77 | -1.04 | -0.72 | 0.79 | 1.84 | -1.80 | -0.36 | 0.62 | 1.38 | -0.09 | -0.08 | 0.32 | 0.09 |
| DaFEx | | 0.94 | -0.12 | 0.67 | 2.62 | -0.15 | -0.08 | -0.17 | -0.35 | -0.43 | 0.41 | -0.51 | -0.53 | 4.03 | 1.18 | 0.44 | 0.16 | -0.56 | -0.46 | -0.57 | -0.03 |
| DISFA | | 0.04 | 0.73 | 0.00 | 0.17 | -0.01 | -0.02 | -0.01 | -0.01 | -0.01 | -0.04 | 0.05 | -0.01 | -0.01 | -0.02 | -0.01 | 0.06 | -0.01 | -0.03 | 2.01 | 0.07 |
| DynEmo | | 0.35 | **9.38** | 0.37 | **9.09** | -1.25 | -2.83 | -0.29 | -0.26 | -0.54 | -0.06 | -0.13 | -0.23 | 0.13 | **8.81** | -0.16 | -0.39 | 0.18 | -0.09 | 0.03 | 0.14 |
| FG-NET | | 4.45 | -0.23 | 0.27 | **8.23** | -0.18 | -1.35 | -0.13 | 0.43 | -0.56 | -0.26 | -0.44 | -1.37 | 0.09 | **14.70** | 0.23 | -0.02 | 0.83 | -0.23 | 0.32 | -1.95 |
| GEMEP | | 0.54 | -0.25 | -0.04 | 0.08 | -0.12 | -0.10 | 0.00 | -0.04 | -0.18 | 0.01 | -0.02 | -0.01 | -0.14 | 0.01 | -0.04 | 0.00 | 0.16 | -0.45 | 0.02 | -0.04 |
| MMI | | 0.63 | **7.34** | -0.39 | **7.92** | -0.31 | -1.68 | -0.35 | -1.15 | -0.47 | -0.93 | -0.68 | -0.50 | 0.02 | **8.32** | -0.34 | 0.45 | -0.02 | -2.02 | 0.62 | -0.25 |
| MPI | | 1.56 | -1.22 | 0.11 | **7.55** | -0.05 | -0.43 | 0.12 | -2.39 | -0.18 | -0.04 | -0.58 | -0.92 | -0.58 | **7.84** | -0.14 | 0.05 | -0.78 | -0.23 | -1.95 | 0.32 |
| STOIC | | 0.35 | -0.07 | -0.33 | 2.11 | -0.14 | -0.22 | -0.34 | -0.26 | -0.24 | -0.03 | -0.20 | -0.09 | -0.06 | 0.07 | -0.14 | -0.09 | 0.84 | 0.19 | 0.00 | -0.01 |
| UT Dallas | | 2.40 | 2.40 | 0.55 | **7.48** | -0.05 | -1.02 | 0.01 | 0.09 | -0.17 | -0.28 | 0.06 | -0.14 | -0.13 | 4.82 | -0.40 | -0.05 | 0.03 | 0.08 | -0.11 | -0.13 |
| **happiness** | |  |  |  |  |  |  |  |  |  |  |  |  |  |  |  |  |  |  |  |  |
| ADFES | | 0.00 | -0.03 | 0.02 | 0.00 | **9.67** | -0.22 | 0.02 | 0.00 | **33.54** | 0.15 | -0.01 | -0.01 | 0.00 | -0.08 | 0.02 | 0.00 | 5.91 | -1.20 | 0.02 | -0.33 |
| BINED | | -2.85 | -1.62 | -9.80 | -4.83 | **7.21** | -3.08 | -4.97 | -4.02 | **22.46** | 4.47 | -6.52 | -1.25 | -3.26 | 5.83 | -5.46 | -0.63 | **14.74** | -3.08 | 0.04 | 2.26 |
| BU-4DFE | | 0.40 | 0.70 | -1.05 | -0.05 | **14.23** | -0.21 | -0.33 | -1.54 | **34.42** | **14.88** | 0.09 | 0.00 | -0.84 | **6.38** | 0.07 | -1.15 | **14.23** | -4.12 | 0.57 | -0.25 |
| CK | | -0.06 | -0.25 | 0.02 | -0.07 | **9.57** | -0.16 | -0.11 | 0.09 | **32.85** | **9.18** | -0.01 | -0.03 | -0.25 | 1.42 | -0.21 | -0.42 | 5.78 | -1.99 | -0.37 | -0.07 |
| D3D-FACS | | -0.43 | -0.12 | -1.48 | -1.83 | 3.13 | 0.83 | -1.30 | -1.33 | **34.47** | **17.53** | -0.62 | -1.61 | -1.93 | **52.14** | -0.59 | -1.09 | 0.02 | 0.06 | 5.83 | -0.04 |
| DaFEx | | -2.04 | 0.59 | -32.32 | -0.77 | **33.94** | **19.63** | -5.88 | -2.64 | **26.03** | **45.44** | -0.51 | -4.85 | -0.35 | 4.26 | -22.93 | -14.59 | **13.61** | -9.14 | **8.52** | -0.59 |
| *Table S2.* (Continued) | | | | | | | | | | | | | | | | | | | | | |
|  | **Action Unit** | | | | | | | | | | | | | | | | | | | | |
| Database | 1 | | 2 | 4 | 5 | 6 | 7 | 9 | 10 | 12 | 14 | 15 | 17 | 18 | 20 | 23 | 24 | 25 | 26 | 28 | 43 |
| DISFA | 6.59 | | -6.10 | -7.24 | -3.38 | **6.93** | -4.87 | -5.09 | -6.53 | **19.29** | 4.28 | -3.48 | -3.59 | -6.00 | 4.68 | -3.67 | 1.20 | 3.84 | -3.07 | 0.66 | -4.59 |
| DynEmo | -1.25 | | -1.21 | -3.50 | -3.84 | **13.35** | -1.30 | -0.65 | 1.33 | **22.71** | 3.40 | -2.72 | -1.50 | -2.97 | 0.00 | -0.20 | -0.55 | **9.63** | -1.73 | -0.11 | -0.62 |
| FG-NET | 0.29 | | 0.21 | -0.19 | -0.08 | **10.70** | -0.80 | -0.50 | -1.15 | **33.04** | 2.42 | -0.13 | -0.54 | -0.18 | 0.81 | 0.17 | -0.89 | **16.73** | -0.14 | -1.57 | 2.26 |
| GEMEP | -2.26 | | 0.39 | -0.32 | -0.34 | 1.32 | -0.20 | 0.03 | -0.12 | 3.29 | 0.00 | -0.21 | -0.06 | -0.57 | -0.42 | -0.15 | -0.02 | 0.40 | -2.38 | 0.04 | -0.45 |
| MMI | -0.57 | | 0.20 | -1.62 | -0.03 | **9.72** | -0.46 | -1.17 | -0.86 | **31.10** | 3.24 | -1.11 | -0.79 | 0.45 | 0.20 | -2.03 | 3.69 | 5.55 | -1.76 | 2.68 | -0.77 |
| MPI | -0.86 | | -3.34 | -3.60 | -2.23 | **7.33** | -4.38 | -4.72 | -3.32 | **26.00** | 3.56 | -2.36 | 0.12 | -3.16 | -0.01 | -1.51 | -1.04 | 4.72 | -2.72 | **7.74** | 0.93 |
| STOIC | -0.43 | | -0.16 | -0.63 | -0.34 | 2.27 | 0.36 | -0.78 | -0.46 | **17.66** | 0.34 | -0.24 | -0.28 | -0.26 | -0.07 | -0.01 | 0.03 | 0.81 | -0.82 | 0.07 | 0.05 |
| UT Dallas | -1.67 | | 0.61 | -4.72 | -2.32 | **10.08** | 0.54 | -3.19 | -1.92 | **27.98** | **9.17** | -0.95 | -1.38 | -2.93 | **6.97** | 3.68 | -0.78 | **13.87** | -1.06 | 3.60 | -0.64 |
| **sadness** |  | |  |  |  |  |  |  |  |  |  |  |  |  |  |  |  |  |  |  |  |
| ADFES | **8.58** | | -2.39 | 2.56 | -0.47 | -0.25 | 0.16 | -0.37 | -0.30 | -0.60 | -0.43 | **13.58** | 1.11 | -0.01 | -0.29 | -0.91 | -1.08 | -0.68 | -0.32 | -0.39 | -0.03 |
| BINED | **18.93** | | -0.15 | 0.75 | -2.36 | -0.53 | 1.17 | -0.06 | -6.22 | -1.09 | 0.54 | **18.66** | -1.18 | 3.73 | -0.35 | 0.62 | 1.46 | -0.19 | -0.73 | 0.28 | 0.80 |
| BU-4DFE | **10.94** | | -4.29 | 2.61 | 0.58 | -0.39 | -0.80 | -0.84 | -1.18 | -1.23 | 1.50 | **10.32** | 5.41 | 5.16 | -0.25 | -1.73 | -1.57 | -1.24 | -0.15 | 0.47 | 3.24 |
| CK | **7.25** | | -1.24 | 2.76 | -0.43 | 0.03 | 0.73 | -0.27 | 0.12 | -0.32 | -0.30 | **12.33** | 4.82 | 4.33 | -0.18 | -0.62 | -2.20 | -0.37 | -0.07 | 0.49 | 5.07 |
| D3D-FACS | **15.92** | | -8.88 | **9.63** | -3.84 | -2.01 | 0.18 | -0.49 | -4.01 | -3.36 | -4.39 | **6.12** | -0.53 | **6.42** | 0.02 | -3.74 | -0.51 | 0.07 | -0.11 | -1.68 | 0.37 |
| DaFEx | **11.41** | | -5.38 | **7.76** | -0.92 | -3.00 | -0.63 | -0.53 | -1.30 | -1.56 | -1.20 | **8.94** | **7.33** | -0.17 | -0.02 | 5.33 | **18.71** | -1.79 | -1.06 | -1.51 | 1.24 |
| DISFA | **6.90** | | -0.20 | -0.01 | -1.13 | -0.38 | -0.76 | -0.02 | -0.36 | -0.02 | 0.02 | **21.27** | -0.79 | 4.18 | **8.40** | -1.00 | 2.59 | -0.10 | 0.18 | 0.90 | 1.36 |
| DynEmo | **13.01** | | -1.30 | 0.69 | -1.46 | -1.78 | 1.53 | -0.76 | -0.61 | -1.74 | 1.10 | **13.24** | **6.61** | 2.77 | -0.11 | 0.86 | -0.81 | -0.99 | -0.29 | 0.21 | 0.66 |
| FG-NET | **17.04** | | -0.57 | 0.17 | -0.32 | -0.80 | 0.58 | -0.01 | -0.54 | -0.01 | -0.93 | **8.97** | **31.76** | 1.15 | 3.84 | -12.94 | 5.54 | -0.31 | -0.32 | -1.78 | **16.14** |
| GEMEP | **10.38** | | -5.84 | 4.62 | -3.64 | -0.70 | 1.53 | 0.02 | -0.57 | -1.33 | 0.02 | 0.11 | 0.62 | 3.39 | 0.34 | 4.18 | 0.41 | -2.63 | -0.97 | -0.36 | -0.34 |
| MMI | **13.78** | | -3.15 | 0.35 | -0.43 | -0.33 | 0.05 | -0.21 | -0.15 | -0.26 | -0.25 | **10.22** | -0.97 | -0.64 | 1.77 | -0.54 | -1.14 | -0.49 | -0.31 | -0.83 | 2.43 |
| MPI | 3.89 | | 0.23 | 0.34 | -0.29 | -0.30 | -0.13 | -0.21 | -0.13 | -0.50 | 0.70 | 0.02 | 0.04 | 0.03 | -0.13 | 0.02 | 0.13 | -0.19 | -0.12 | -0.14 | 0.46 |
| STOIC | **15.33** | | -0.48 | 0.07 | -0.19 | 0.25 | 0.36 | -0.29 | -0.18 | -0.46 | -0.33 | **11.50** | 3.06 | 0.01 | **8.25** | -0.13 | -0.06 | -0.34 | 0.15 | -0.29 | -0.21 |
| UT Dallas | **18.56** | | -2.51 | 4.14 | 0.29 | -0.55 | 0.33 | -0.48 | -1.97 | -0.53 | -0.14 | **8.32** | **6.71** | 4.91 | 0.21 | -1.21 | 0.48 | -0.20 | 0.17 | -0.17 | 2.43 |
| **surprise** |  | |  |  |  |  |  |  |  |  |  |  |  |  |  |  |  |  |  |  |  |
| ADFES | 0.59 | | **11.29** | -0.12 | -0.58 | -0.90 | -0.73 | -0.09 | -0.11 | -0.94 | -0.03 | -0.10 | -0.27 | 0.02 | -1.18 | -0.04 | 0.00 | **14.78** | **10.98** | 0.02 | 0.59 |
| BINED | -0.32 | | **7.09** | 0.10 | -0.01 | -0.05 | -0.41 | 0.09 | -0.17 | -0.01 | -0.03 | -0.14 | 0.14 | 0.39 | -1.24 | -0.09 | -0.07 | 0.02 | **13.05** | 0.21 | 1.11 |

| *Table S2.* (Continued) | | | | | | | | | | | | | | | | | | | | |
| --- | --- | --- | --- | --- | --- | --- | --- | --- | --- | --- | --- | --- | --- | --- | --- | --- | --- | --- | --- | --- |
|  | **Action Unit** | | | | | | | | | | | | | | | | | | | |
| Database | 1 | 2 | 4 | 5 | 6 | 7 | 9 | 10 | 12 | 14 | 15 | 17 | 18 | 20 | 23 | 24 | 25 | 26 | 28 | 43 |
| BU-4DFE | 4.92 | **14.16** | -0.45 | 5.51 | -0.13 | -0.91 | 0.17 | -0.44 | -0.34 | -2.86 | -0.25 | -0.19 | -0.28 | -0.76 | -0.03 | -0.10 | 2.75 | **13.71** | 0.11 | 0.95 |
| CK | 5.13 | **16.32** | 0.00 | 2.21 | -1.37 | -0.22 | 0.26 | -0.23 | -0.63 | -0.29 | -0.05 | 0.04 | -0.48 | -1.75 | 0.05 | 0.00 | **18.62** | **10.28** | -0.15 | -0.53 |
| D3D-FACS | -0.12 | 1.90 | -0.11 | 0.53 | -0.35 | -0.06 | -0.06 | -0.15 | -0.13 | -0.16 | 0.27 | -0.07 | 0.18 | -0.09 | 0.05 | -0.36 | 0.00 | 0.02 | -0.18 | 0.14 |
| DaFEx | 4.85 | **12.99** | -2.43 | **6.79** | -5.67 | -13.30 | -0.39 | -0.23 | -0.79 | -4.12 | -0.85 | -1.79 | -3.02 | -0.52 | -1.93 | -0.97 | -3.63 | **25.30** | -0.96 | **6.05** |
| DISFA | -0.78 | **9.28** | -0.04 | **64.58** | 0.01 | -5.12 | 0.04 | -0.08 | -0.05 | -0.12 | -0.16 | 0.34 | 0.25 | -0.24 | 0.20 | 0.13 | 0.06 | 4.36 | -0.56 | 0.15 |
| DynEmo | 0.15 | 0.73 | -0.20 | 3.23 | -0.46 | -0.53 | -0.08 | -0.31 | -0.33 | -0.29 | -0.05 | -0.07 | 0.01 | -0.58 | -0.13 | -0.20 | 0.50 | **9.75** | -0.11 | 1.03 |
| FG-NET | -1.80 | **11.62** | 0.15 | 2.65 | -0.13 | -0.17 | 0.19 | -0.04 | -0.22 | -0.32 | -0.08 | -2.96 | 0.69 | -1.85 | 0.69 | -0.15 | 3.49 | **11.89** | 0.19 | -1.94 |
| GEMEP | 0.48 | 4.38 | -0.80 | 0.79 | -1.10 | -1.15 | -0.04 | -0.47 | -1.52 | -0.48 | -0.82 | -0.21 | -1.40 | -2.46 | -0.80 | -0.35 | 0.74 | **7.91** | -0.77 | 1.27 |
| MMI | 2.78 | 1.85 | -0.22 | -0.15 | -0.17 | -0.25 | -0.05 | -0.17 | -0.33 | -0.33 | -0.10 | -0.14 | -0.45 | -0.36 | -0.09 | -0.57 | 2.45 | **61.00** | -0.69 | -0.12 |
| MPI | 0.66 | **14.84** | -0.25 | 0.79 | -0.29 | -0.54 | -0.16 | -0.20 | -0.61 | -0.33 | -0.26 | -0.46 | 0.32 | -0.44 | -0.20 | -0.21 | 0.18 | **11.81** | -0.41 | 0.10 |
| STOIC | -0.22 | 1.22 | -0.87 | -0.70 | -0.37 | -0.51 | -0.60 | -0.70 | -0.56 | -0.09 | -0.46 | -0.19 | -0.03 | -0.56 | -0.23 | -0.23 | 5.78 | **6.02** | 0.04 | 0.04 |
| UT Dallas | 0.27 | **6.45** | 0.04 | 1.44 | -0.03 | -0.68 | 0.03 | -0.24 | -0.21 | -0.88 | -0.11 | -0.08 | 0.11 | -1.04 | -0.54 | -0.16 | 0.99 | **8.33** | -0.13 | 1.04 |

*Note.* Regression coefficients (β) > 6.0 are printed in bold. The prior of p_0_ were happiness = 1, surprise = 3, anger = 4, sadness = 2, disgust = 1, fear = 5. See Table S2 for results per database.
